# Recent Advances in Nucleic Acid Targeting Probes and Supramolecular Constructs Based on Pyrene-Modified Oligonucleotides

**DOI:** 10.3390/molecules22122108

**Published:** 2017-11-30

**Authors:** Olga A. Krasheninina, Darya S. Novopashina, Evgeny K. Apartsin, Alya G. Venyaminova

**Affiliations:** Institute of Chemical Biology and Fundamental Medicine SB RAS, Acad. Lavrentiev Ave. 8, Novosibirsk 630090, Russia; danov@niboch.nsc.ru (D.S.N.); eka@niboch.nsc.ru (E.K.A.); ven@niboch.nsc.ru (A.G.V.)

**Keywords:** oligonucleotides, pyrene, fluorescent probes, nucleic acids, SNP detection, aptasensors, G-quadruplexes, TINA, Invader probes, nanoconstructions, supramolecular assemblies

## Abstract

In this review, we summarize the recent advances in the use of pyrene-modified oligonucleotides as a platform for functional nucleic acid-based constructs. Pyrene is of special interest for the development of nucleic acid-based tools due to its unique fluorescent properties (sensitivity of fluorescence to the microenvironment, ability to form excimers and exciplexes, long fluorescence lifetime, high quantum yield), ability to intercalate into the nucleic acid duplex, to act as a π-π-stacking (including anchoring) moiety, and others. These properties of pyrene have been used to construct novel sensitive fluorescent probes for the sequence-specific detection of nucleic acids and the discrimination of single nucleotide polymorphisms (SNPs), aptamer-based biosensors, agents for binding of double-stranded DNAs, and building blocks for supramolecular complexes. Special attention is paid to the influence of the design of pyrene-modified oligonucleotides on their properties, i.e., the structure-function relationships. The perspectives for the applications of pyrene-modified oligonucleotides in biomolecular studies, diagnostics, and nanotechnology are discussed.

## 1. Introduction

Pyrene-modified oligonucleotides have gained much attention as tools for nucleic acid research, diagnostics, and nanotechnology. Specific examples of pyrene-modified oligonucleotides reviewed here include sensitive fluorescent probes for the sequence-specific detection of nucleic acids (NAs) and discrimination of single nucleotide polymorphisms (SNPs), aptamer-based biosensors for the detection of a wide range of various targets, agents for binding of dsDNAs, building units of supramolecular complexes, and other applications. These simple synthetically-accessible molecules integrate an excellent recognition capability of oligonucleotides and ability of pyrene to act as microenvironment-sensitive fluorescent label [1,2,3,4], excimer- or exciplex-generating molecule [5,6,7,8,9,10,11,12,13,14,15], NA complex intercalator [16,17,18,19], or π-π-staking hydrophobic moiety [20,21]. It is worth noting that these functions of pyrene are especially brightly expressed in the NA context. For instance, pyrene displays a different fluorescence output signal being displaced in the minor or major groove of the NA duplex compared to the intercalating mode, which is widely exploited in a number of studies (see, for instance, [18]). Electronic coupling of pyrene to a heterocyclic moiety (e.g., nucleobase) or extending of π-system of pyrene through alkyne substitution results in bathochromic shifts in absorption and fluorescence spectra [4,22,23,24,25,26,27]. One of the most notable properties of pyrene and some of its derivatives, is the ability to form excited dimers and complexes, so-called excimers and exciplexes. The excimer is formed when a photoexcited pyrene molecule is in close proximity with a non-excited one, resulting in appearance of a new red-shifted signal relative to monomer. For efficient excimer formation, pyrene molecules must be oriented parallel with an interplanar distance of 3–4 Å, which almost corresponds to the length of one base pair along the DNA duplex. Additionally, an excited pyrene molecule can also act as a donor of fluorescence when located in close proximity to a relevant acceptor fluorophore in the ground state (Förster resonance energy transfer, FRET). Careful design of fluorescent systems based on FRET allows the production of new probes possessing unique signal properties, e.g., different Stokes shifts and increased intensity [11,12,13,14,15,28,29,30,31,32]. Thus, since relative positions of pyrenes strongly affect excimer or exciplex formation, this can be used to obtain precise information about the nucleic acid structure (Section 2, Fluorescent Biosensors). The ability of pyrene moieties to intercalate into a nucleic acid duplex, as a result of a comparable staking area (~184 Å) to that of natural base pairs (~200 Å), and to form stacking pairs, have motivated scientists to develop agents stabilizing NA complexes (Section 3, Agents for the Targeting of dsDNAs) and assembling sophisticated supramolecular oligonucleotide-based constructions (Section 4, Supramolecular Assemblies). Moreover, pyrene can be a structural element of functional oligonucleotides (for instance, ribozymes) improving their recognition properties (Section 5). Along with the discussion of the applications of pyrene-modified oligonucleotides, the review will highlight the influence of their design on the resulting properties.

## 2. Fluorescent Biosensors

### 2.1. Fluorescent Probes for the Detection of RNA and DNA

Currently, one of the most reliable methods for the detection and study of functions and structural features of NAs, detection of single nucleotide polymorphisms (SNPs), quantification of NA in vitro, and visualization of NA in cells is the use of fluorescently-labeled oligonucleotide probes complementary to short regions of full-size NA targets. At the moment, several types of fluorescent probes based on pyrene-labeled oligonucleotides, which display strong hybridization-induced changes and a high degree of selectivity of probes relative to the type of NA target, have been reported.

**Figure 1:**
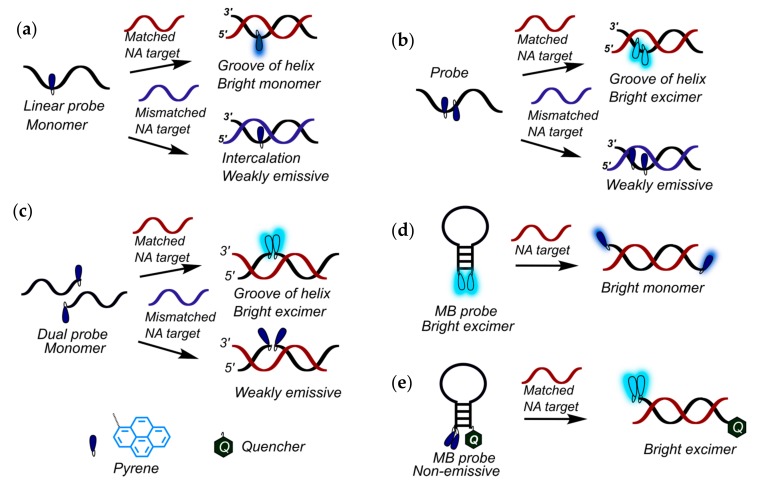
Schematic representation of principles of some pyrene-modified fluorescent probes: linear probes (**a**,**b**); dual probes (**c**); and molecular beacons (MBs) (**d**,**e**).

Fluorescent probes based on pyrene-modified oligonucleotides can be classified into several types: linear probes (Figure 1a,b), dual (or tandem) probes (Figure 1c), molecular beacons (MBs) (Figure 1d,e), including excimer- or exciplex-forming probes, and base-discriminating probes. In each case, the probes are rationally designed to display significant hybridization-induced changes in fluorescence emission, for instance, pronounced increases/decreases in pyrene monomer fluorescence or effective formation/disappearance of a red-shifted signal of excimer or exciplex fluorescence.

It is well known that fluorescence of the pyrene moiety attached to oligonucleotide is very sensitive even to subtle changes of the local environment. The signal may have a high level when the pyrene moiety is in a non-quenching environment of helix grooves or can be quenched through interactions with nearby nucleobases. Thus, the degree of the fluorescence signal changes strongly depends on the disposition of pyrene in the resulting hybrid probe-target duplex. In general, more efficient overlap of pyrene moieties with nearby nucleobases and, consequently, more essential fluorescence signal reduction occurs within a B-type duplex (DNA:DNA). Intercalation of pyrene residues into the more compact A-type duplex (RNA:RNA) is less favorable, which makes pyrene a very valuable label for RNA-specific probes. At the same time, some types of modifications provide a possibility of the positional control of the pyrene moieties in nucleic acids complexes [4,33]. Thus, to obtain pyrene-modified probes, with the significant target hybridization-induced output signal changes, it is necessary to design the probes taking into account structural features of the NA target, the pyrene fluorescence generating units (i.e., type of modification, length and type of linker between pyrene and the oligonucleotide), the type of the oligonucleotide part of the probe, and the resulting hybrid complex. Previously, the physicochemical properties of pyrene and its derivatives in NA contexts, a diversity of pyrene-modified oligonucleotide probes, their hybridization properties and some specific design rules for the positioning of pyrene-modified monomers for successful design of the various fluorescent probes were well described in some other related reviews published several years ago [4,7,9,10,33,34,35]. Herein, we focus only on the recently published works that were not covered in the above-mentioned reviews. 

**Figure 2:**
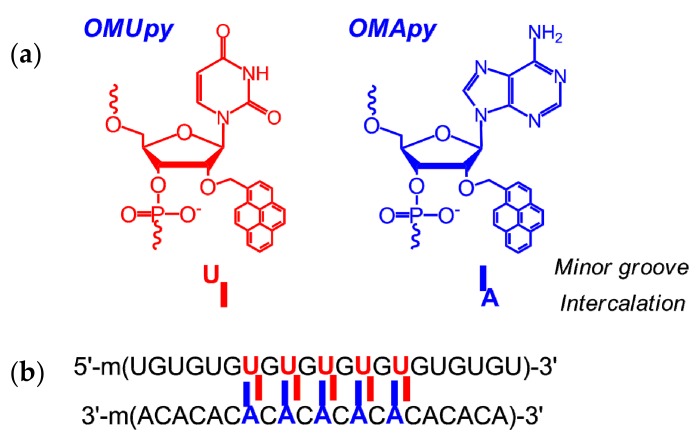
(**a**) Chemical structures of 2′-*O*-(pyrene-1-yl)methyluridine and 2′-*O*-(pyrene-1-yl)methyladenosine monomers (**OMUpy** and **OMApy**) and the disposition of pyrene in the hybrid probe-target duplex; and (**b**) an example of 2′-*O*-methyl RNA duplex with a pyrene zipper array [46].

Yamana, Murakami, and coauthors developed and studied in detail unambiguously sensitive RNA-targeted oligonucleotide probes modified with one or more insertions of 2′-*O*-(pyrene-1-yl)methyluridine monomer (**OMUpy**) (Figure 2a) [36,37,38,39,40,41,42,43,44,45]. The authors showed that linear 2′-*O*-methyl RNA probes (Figure 1b) modified with two consecutive pyrene-functionalized **OMUpy** monomers and flanked by a 3′-pyrimidine or guanine moiety are highly RNA-specific and display exceptional increases in pyrene excimer fluorescence when hybridized to complementary RNA [38,39,42,43,44,45]. Their studies indicated that the pyrene moiety of the RNA:RNA duplexes are located in a minor groove outside of the duplex, whereas if the pyrene moiety incorporated into the DNA:DNA duplex it intercalates into the double helix (as confirmed by NMR studies) [18]. 

Owing to these unique predictable properties, the 2′-*O*-(pyrene-1-yl)methyl-modified nucleotide monomers have been used as components of highly sensitive probes in a number of fluorescent approaches to RNA detection [36,37,38,39,40,41,42,43,44,45,47], as SNP-discriminating RNA-specific fluorescent probes [48,49], as key components in highly-fluorescent pyrene π-stack arrays on RNA (Figure 2b) (Section 2.3.6) [46,50,51,52,53,54,55], and also as intercalating units efficiently stabilizing DNA:DNA duplexes (Section 3.2) [56,57,58,59,60,61,62,63]. The properties and applications of the oligonucleotides comprising 2′-*O*-(pyren-1-yl)methylribonucleotides monomers have been discussed in detail in the just published review [64].

**Figure 3:**
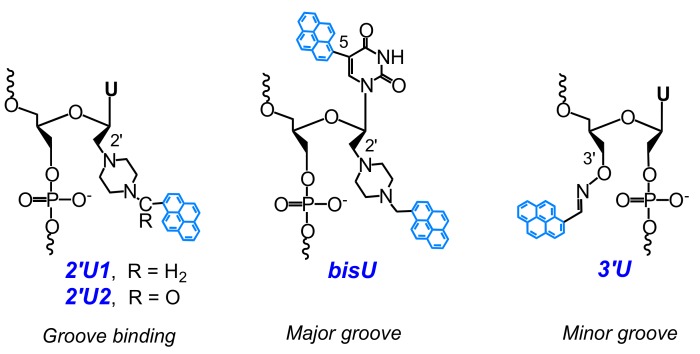
Chemical structures of pyrene-modified unlocked nucleic acid (UNA) monomers: 2′-pyrene-UNA monomers (**2′U1** and **2′U2**) [65,66], bis-pyrene-UNA monomer (**bisU**) [67], and 3′-pyrene-UNA monomer (**3’U**), respectively, and the disposition of pyrene in hybrid probe-target duplexes [68]. U = uracil-1-yl.

Wengel and coworkers have developed a series of hybridization probes comprising derivatives of unlocked nucleic acid (UNA) monomers bearing a pyrene moiety attached through a piperazine ring to the C2′-atom of UNA (monomers **2′U1** and **2′U2**) (Figure 3) [65]. They have found that the incorporation of the 2′-pyrene-UNA monomers **2′U1** and **2′U2** increase duplex stability compared with UNA monomers, most probably due to the groove binding mode of interaction of pyrene with the NA duplex. The 2′-pyrene-UNA modified oligonucleotides demonstrated increased fluorescence emission intensities upon hybridization to DNA. Intriguingly, they observed a high intensity of pyrene excimer emission for single-stranded oligonucleotides containing three 2′-pyrene-UNA modifications **2′U1** and **2′U2**, which disappeared after hybridization to DNA. Later, in the frame of other work, they used these observations to construct an excimer-forming quencher-free molecular beacon modified by strategically positioned 2′-pyrene-UNA monomers **2′U1** [66]. Then Wengel and coauthors introduced oligonucleotide probes modified with bis-pyrene-UNA monomer **bisU** (Figure 3) bearing one pyrene group directly attached to the 5-position of uracil and the second pyrene to the 2′-position through a piperazino linker [67]. The incorporation of the bis-pyrene-UNA monomers (**bisU**) led to the thermal destabilization of both types of duplexes (probe:DNA and probe:RNA), presumably due to the interactions of pyrene moieties with major grooves. The probes comprising two **bisU** monomers display low pyrene monomer fluorescence in bulge-containing duplexes, high pyrene monomer fluorescence in duplexes, and exciplex emission of 5-(pyrenyl)uracil with pyrene in the single-stranded form. Thus, the single-stranded form, duplex, and bulge-containing duplex can be easily distinguished simply by measuring the fluorescence at 380 nm (monomer) and 450 nm (exciplex) [67]. More recently, the same group of authors have proposed a new 3′-*O*-pyren-1-ylmethanimino UNA monomer (**3’U**) (Figure 3) that was incorporated one, two, or three times into 21-mer DNA or 2′-*O*-methyl RNA [68]. The **3’U** monomer, when incorporated into DNA or into 2′-*O*-methyl RNA 21-mers, increased the thermal stability of duplexes with a DNA complement. Pyrene excimer fluorescence emission was registered for single-stranded probes with two or three incorporations of the **3’U** monomer. Hybridization with a complementary DNA resulted in the disappearance of excimer emission and an increase of monomer emission, which confirms the disposition of pyrene moieties in the minor grooves.

A large diversity of the pyrene-modified nucleotide monomers obtained through presynthetic [69,70,71,72,73,74,75] or postsynthetic [76,77,78,79,80,81] conjugation of pyrene derivatives with modified nucleotides or oligonucleotides via Cu(I)-catalyzed azide alkyne Huisgen 1,3-dipolar cycloaddition (CuAAC) has been reported over the past several years. 

**Figure 4:**
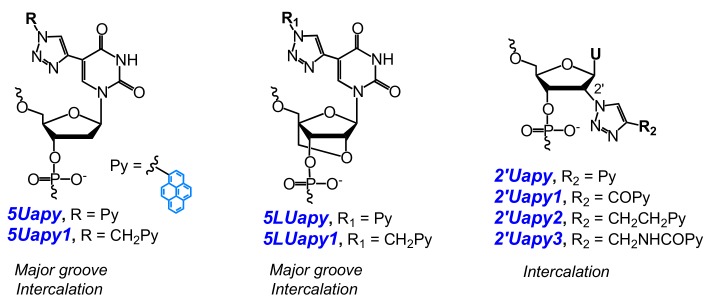
Structures of the pyrene-triazole nucleotide monomers studied in [69,70,71,74] and the disposition of pyrene in the hybrid probe-target duplex. U = uracil-1-yl.

For instance, Hrdlicka and coauthors used CuAAC in presynthetic strategy for the preparation of triazole-linked C5- or C2′-pyrene-functionalized monomers shown in Figure 4 [69,70,71,74]. An incorporation of C5-pyrene-functionalized monomers **5Uapy**, **5Uapy1**, and their LNA analogues **5LUapy** and **5LUapy1** into DNA showed trends to destabilize nucleic acid duplexes [69,71,74]. C5-pyrene-functionalized oligonucleotide probes demonstrated significant hybridization-induced increases in fluorescence spectra and enabled efficient fluorescence discrimination of SNPs for both types of NA targets. The pyrene moiety is presumably positioned in the major groove. It is highly emissive when the probes are bound to fully complementary targets whereas, in the case of mismatched targets, pyrene tends to intercalate into the duplex [69,74]. These remarkable properties of C5-pyrene-functionalized monomers were utilized to construct a probe with four consecutive incorporations of monomer **5Uapy** in the center, which possesses high affinity and specificity to RNA targets due to the formation of the pyrene π-stack array in the major groove of the duplex. This probe demonstrates exceptional SNP-discriminating properties upon thermal melting of the duplexes [71]. Oligodeoxyribonucleotides modified with C2′-pyrene-functionalized monomers **2′Uapy**–**2′Uapy3** (Figure 4) displayed slightly decreased thermal stability of NA duplexes, and high affinity toward DNA targets with the abasic site located opposite to the monomer site. They are also universal hybridization probes, i.e., probes that have nearly identical affinities toward fully-matched and mismatched RNA/DNA. Physicochemical studies suggest that the pyrene moiety is intercalating into the duplex, whereby the opposing nucleotide is pushed out [70].

**Figure 5:**
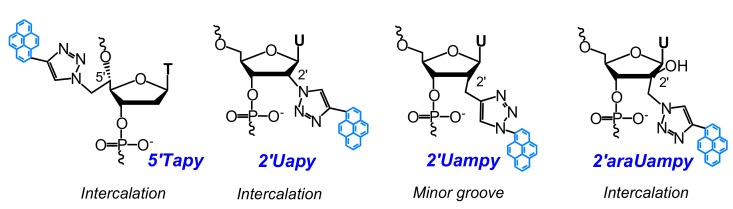
Pyrene-triazole nucleotide monomers studied in [72] (**5’Tapy**, **2′Uapy** and **2′Uampy**) and in [75] (**2′araUampy**) and disposition of pyrene in the hybrid probe-target duplex. T = thymine-1-yl, U = uracil-1-yl.

At the same time, Nielsen and coauthors have introduced a series of pyrene-modified nucleosides (Figure 5) accomplished with the use of the CuAAC reaction [72,75].

All the monomers are found to have different effect on properties of formed nucleic acid duplexes. Based on the quenching of pyrene fluorescence upon hybridization, as well as the increase in duplex thermal stability, the authors concluded that the pyrene moiety of the **5’Tapy** monomer in duplexes of the DNA probe with DNA and RNA, targets presumably adopt an intercalating binding mode. In case of the **2′Uapy** monomer linker between pyrene and ribose being rigid, resulting in the tendency of pyrene to intercalate into the DNA:DNA duplexes, however, the thermal stability of the duplexes decreases. The pyrene moiety of the **2′Uampy** monomer is most probably partly positioned in the minor groove, as the effect on fluorescence intensity varies on the nucleobase sequence, but in most cases leading to an increase in duplex stability [72]. On the other hand, the pyrene attached to the 2′-position of arabino-uridine **2′araUampy** tends to intercalate into DNA:DNA duplexes, thus strongly stabilizing DNA duplexes. The oligonucleotide probe comprising two consecutive **2′araUampy** monomers in the center displays a four-fold increase in pyrene excimer fluorescence upon hybridization with RNA targets, but not with DNA targets [75]. When these monomers were introduced in bulged duplexes or in the core of three-way junctions, thermal stabilization was observed for all four monomers [72,75]. The findings show the potential of the pyrene-triazole nucleotides in various applications as a basis of new diagnostic probes and nucleic acid targeting agents.

Krasheninina et al. developed and studied three variants of pyrene excimer-forming 2′-*O*-methyl RNA probes, namely linear probes, dual probes, and MBs for the detection of RNA [82,83,84,85]. The probes were obtained via simple and efficient postsynthetic labeling method as described in [86,87].

**Figure 6:**
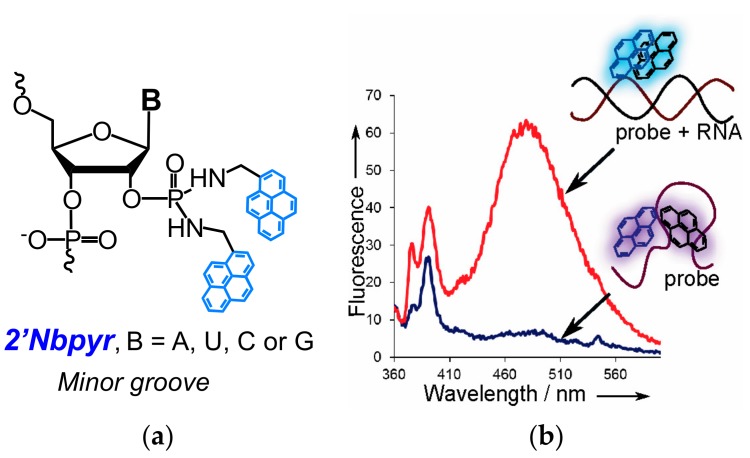
(**a**) 2′-Bispyrene-modified nucleotide monomers studied in [82,83] (**2′Nbpyr**) and the disposition of pyrene in the hybrid probe-target duplex; (**b**) Fluorescence emission spectra of linear probes containing one **2′Nbpyr** monomer (B: cytidin-1-yl). A: adenin-9-yl, U: uracil-1-yl, C: cytidin-1-yl, and G: guanin-9-yl.

The first variant, linear 2′-*O*-methyl RNA probes containing one to three 2′-bispyrene-modified monomers (Figure 6) were found to be RNA-specific fluorescent probes [82,83]. In general, the probes, being much more synthetically accessible, demonstrated similar properties that were observed for other variants of probes comprising monomers with pyrene introduced to the 2′-position of ribose through a short linker, indicating the disposition of pyrenes in the minor groove of the RNA duplex. Namely, introduction of the **2′Nbpyr** monomers (Figure 6a) decreased the thermal stability of duplex probes with RNA targets and, depending on the nucleotide context, had different effects on duplexes with DNA, whereas significant increases (up to 21-fold) in pyrene excimer fluorescence intensity were observed upon binding of most of the 2′-bispyrene-modified probes with complementary RNA (Figure 6b), but not with DNA. We found that the fluorescent properties of the probes upon hybridization with RNA depended on the nucleotide context of the modified ribonucleoside and were optimal in the case of 3′-flanking CC, CG, or UC dinucleotide units [83].

In comparison with other described types of oligonucleotide-based excimer- and exciplex-forming probes, the dual probes are characterized by their improved selectivity (Figure 1c) [88]. There should be no specific signal unless both adjacent components of the probe hybridize with the NA target, providing the proper positioning of fluorophores necessary for the excimer or exciplex formation. Previously, the potential of pyrene-labeled dual probes was demonstrated for the detection of RNA in cellular extracts by means of time-resolved fluorescence spectroscopy [89], and for discrimination of SNPs in NA with the use of excimer- [90,91] or exciplex-generating probes [13,14].

**Figure 7:**
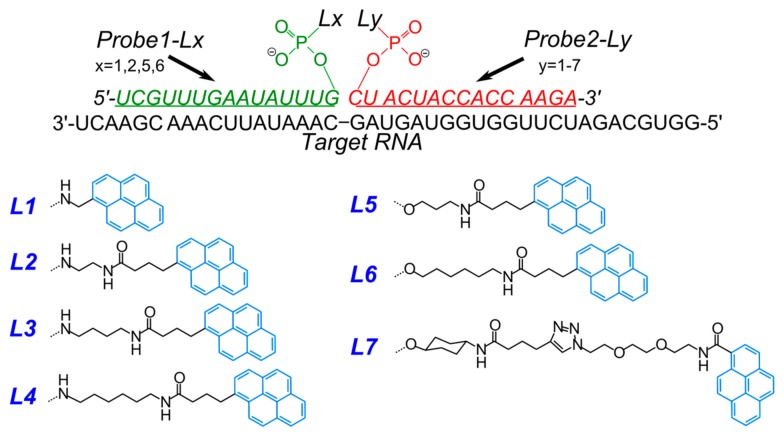
Schematic representation of the RNA targeted excimer-forming dual probes studied in [84]. Probe1-Lx and Probe2-Ly represent components of dual 2′-*O*-methyl RNA probes bearing a pyrene residue attached to the 3′- and 5′-end (**Lx** and **Ly**, respectively) via linkers of different lengths and structure.

In [84] new pyrene excimer-forming dual probes for the visualization of intracellular RNA were studied. The authors constructed 28 variants of dual probes based on 2′-*O*-methyl RNA with linkers of different structure and length between the pyrene moiety and ribose (Figure 7) and studied their hybridization and spectral properties. They found that the shortest linkers **L1** provide more intense excimer emission (at ~480 nm) of RNA-bound probes (signal to background ratios were up to 33); particularly, the length of the linker arm of the 3′-component of dual probes plays a key role in formation of pyrene excimer.

**Figure 8:**
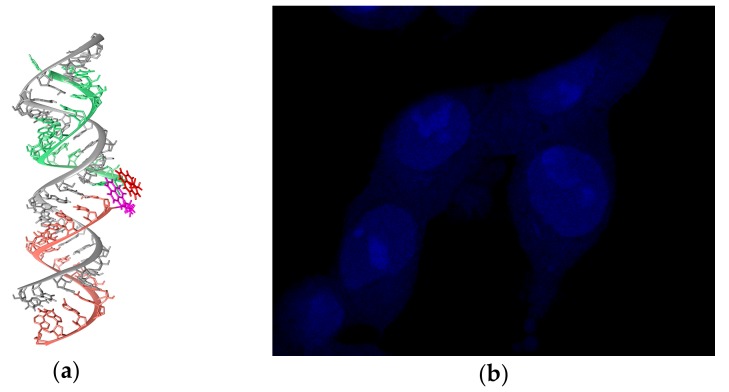
(**a**) Representative structure obtained from the molecular dynamics simulation for complex of Probe1-L1 and Probe2-L5 with an RNA target; (**b**) Visualization of the dual pyrene-labeled 2′-*O*-methyl RNA probes (Probe1-L1 and Probe2-L1) hybridized to a human 28S ribosomal RNA target (GenBank ID 337381, fragment 2326–2357 nt) in fixed HEK293 Phoenix cells [84].

MD simulations of complexes of the dual probes with RNA target are in agreement with the obtained fluorescence spectroscopy data for the corresponding duplexes (Figure 8a). Furthermore, the feasibility of the use of the optimized dual probes for visualization of intracellular 28S rRNA was demonstrated (Figure 8b) [84].

**Figure 9:**
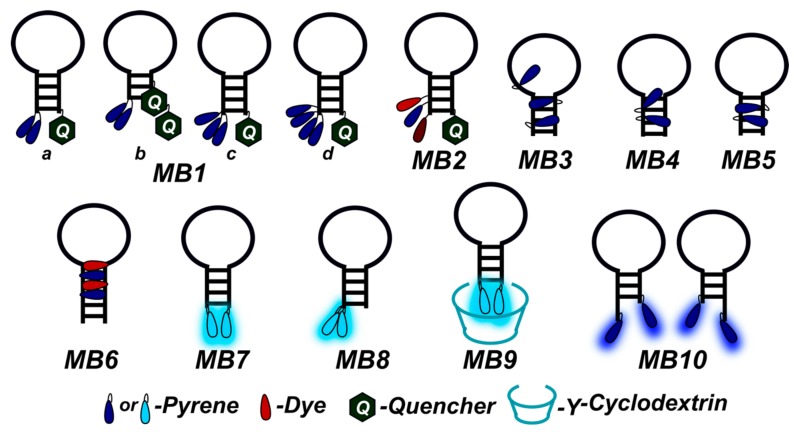
Pyrene-modified molecular beacons.

Light-up systems based on molecular beacons (MBs), i.e., structured pyrene-modified oligonucleotide probes, are powerful tools for sensitive fluorescence detection of a broad spectrum of different targets [66,85,92,93,94,95,96,97]. Originally, MBs are oligonucleotides forming a stem-loop-structure and labeled by a fluorescent group and a quencher at opposite ends of the probe to display an on/off output pattern [98]. Due to the remarkable property of pyrene fluorophores to form excimers and exciplexes with pronounced changes in output fluorescence signals, pyrene-modified MBs have been well developed. To obtain MBs with improved emission characteristics and specificity, several interesting design decisions have been proposed (see, for instance, [29,30,66,85,92,94,95,99,100,101,102,103,104,105,106,107,108,109,110,111]; some of them are illustrated in Figure 9), among them are excimer-forming MBs containing a quencher (for instance, types **MB1a** [85,100,105], **MB1b** [105], **MB1c** and **MB1d** [100], and **MB2** [30]) and numerous quencher-free MBs (for instance, types **MB3** [66], **MB4** [94], **MB5** [29,101], **MB6** [109], **MB7** [106,108], and **MB8** [107]), including systems that utilize γ-cyclodextrin or hybridization chain reaction-mediated signal amplification (types **MB9** [95,111] and **MB10** [104]).

**Figure 10:**
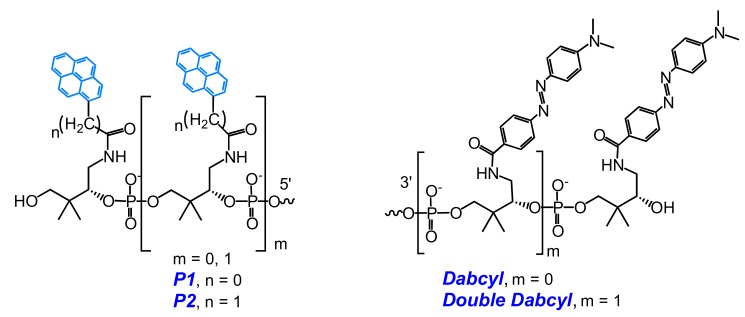
Structures of non-nucleoside pyrene units **P1** and **P2** and quencher units **Dabcyl** and **Double Dabcyl [105]**.

Recently, Korshun and coauthors developed a number of new MBs (types **MB1a** and **MB1b**) labeled with one or two non-nucleoside pyrene monomers **P1** and/or **P2** and one or two moieties of Dabcyl quencher (units **Dabcyl** and **Double Dabcyl**) [105] (Figure 10). 

They have found that the use of **Double Dabcyl** quencher does not result in any benefit in fluorescence properties of the MBs; in contrast, in some cases the excimer fluorescence signal was even higher than for the single **Dabcyl** quencher. Excimer fluorescence maxima of the MBs with different compositions of pyrene units varied from 475 nm to 510 nm; at that, the probes displayed a high excimer/monomer emission ratio and a considerable increase in excimer fluorescence upon hybridization to a complementary DNA target [105].

**Figure 11:**
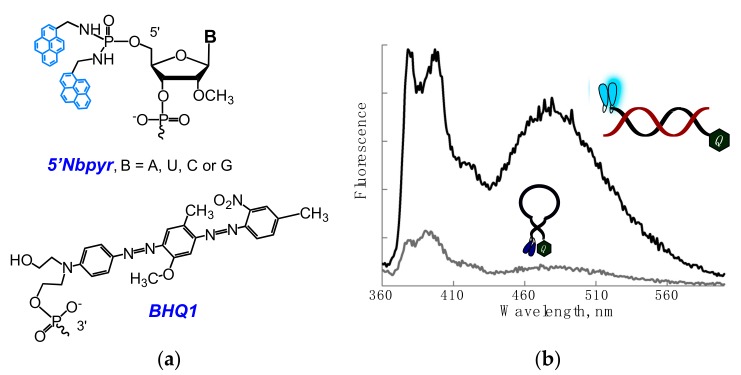
(**a**) Structures of 5′-bispyrene-modified nucleotide monomers **5**′**Nbpyr** and the quencher of fluorescence, **BHQ1**; (**b**) The fluorescence emission spectra of MB containing **5**′**Nbpyr** monomer (B: guanin-9-yl) [85]. A: adenin-9-yl, U: uracil-1-yl, C: cytidin-1-yl, and G: guanin-9-yl.

New excimer-forming 5′-bispyrene-modified molecular beacons (type **MB1a**) for the detection of RNA targets were designed in [85]. The probes are 2′-*O*-methyl RNAs containing a 5′-bispyrenylmethylphosphorodiamidate group (bispyrene group) at the 5′-end (**5′Nbpyr** monomer) and a fluorescence quencher (**BHQ1**) at the 3′-end (Figure 11a). The incorporation of a pyrene moiety was performed with the use of simple and efficient postsynthetic method of modification via the Mukaiyama reaction with nucleophilic catalysis according to [86,87].

A comparative study of the fluorescent properties of the probes having different distances between the 5′-bispyrene group and the target RNA upon the formation of the hybridization complex demonstrated that the probes with the bispyrene group located in close proximity to the duplex exhibit the greatest excimer fluorescence upon binding to a complementary 43-nt target RNA (Figure 11b), in contrast to the probes with the 5′-bispyrene group at dangling ends. The observations indicate a potential of the MBs as SNP discriminating probes. Furthermore, the feasibility of the use of the optimized MBs for visualization of intracellular 28S rRNA was demonstrated [85].

Wengel and coauthors developed a new excimer-forming quencher-free molecular beacon (Figure 9, type **MB3**) modified by strategically-positioned 2′-pyrene-UNA **2′U1** monomers (Figure 3) [66]. Hybridization of the MBs with a complementary RNA target resulted in up to a 10-fold pyrene excimer emission increase, which allowed the possibility of detection of the circular RNA target ciRS-7 RNA in living cells to be demonstrated with the use of probes of optimal design.

**Figure 12:**
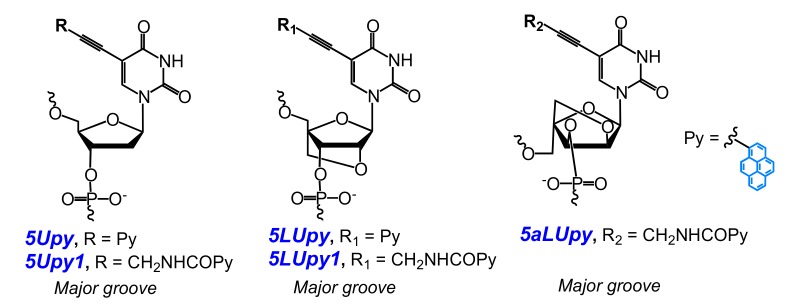
Structures of nucleobase-modified monomers studied in [74,113,114,115,116] and the disposition of pyrene in the hybrid probe-target duplex.

Kim and coauthors developed a new quencher-free MB (type **MB5**) that allow the sensitive probing of special CAG/CTG trinucleotide repeats, which, being extended in the genome, can result in serious hereditary diseases [29]. They designed a series of six MBs featuring two **5Upy** monomers (Figure 12) in symmetric positions within the (CTG)_10_ framework, based on hypothesis that (CTG)_10_ usually forms a hairpin structure and has a tendency of pyrenes of **5Upy** to be quenched by self-staking. The MB of the optimal structure demonstrated a significant hybridization-induced increase in fluorescence intensity mediated by the disruption of the stacking of their **5Upy** units.

### 2.2. Base-Discriminating Probes

The development of tools for sensitive discrimination of NA-containing point mutations (SNP, one nucleotide insertion, or deletion) is a problem of current importance [112]. A diversity of methods for detection of point mutations is based on the hybridization of fluorescent oligonucleotide probes to its complementary NA target to generate different fluorescent signals depending on the nature of opposite nucleotides. In general, the probes for discrimination of point mutations are designed to display significant hybridization-induced changes in fluorescence emission when targeted to a fully-matched NA target and moderate changes or complete quenching of fluorescence upon binding with a mismatched NA target. Thus, the type and the position of the pyrene modification in the probes have to provide different binding modes of pyrene in NA duplexes depending on the nature of opposite and neighboring nucleobases.

**Figure 13:**
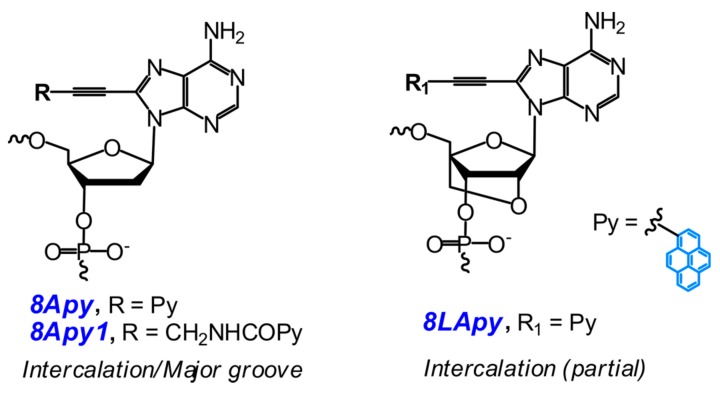
Structures of nucleobase-modified monomers studied in [115,117] and the disposition of pyrene in the hybrid probe-target duplex.

Hrdlicka and coauthors studied a series of nucleobase-modified monomers comprising pyrene attached through alkynyl linkers (Figure 12 and Figure 13). They hypothesized that the introduction of the pyrene moiety via rigid linkers would result in the disposition of pyrene in the major groove of the NA duplex of the modified probe with the target and provide them with SNP discrimination properties. The probes (Figure 1a) comprising C5-alkynyl-functionalized monomers based on DNA (**5Upy** and **5Upy1**) [113,115], LNA (**5LUpy** and **5LUpy1**) [74,113], or α-L-LNA (**5Upy** and **5aLUpy**) (Figure 12) [113,114] have shown almost the same tendencies in physicochemical properties, such as an increase in fluorescence intensity upon hybridization to the NA target and excellent fluorescent discrimination of mismatched DNA targets, although in the case of the probes modified by LNA monomer **5LUpy1** these characteristics were more pronounced.

In contrast, C8-alkynyl-functionalized monomers **8Apy**, **8Apy1**, and **8LApy** (Figure 13) were shown to be detrimental to the binding affinity and specificity [115,117]. Significant improvement of binding affinities and fluorescent discrimination of SNPs in DNA targets of the probes containing monomers based on DNA **5Upy** and **5Upy1** have been achieved by the introduction of LNA nucleotides as direct neighbors flanking the C5-alkynyl-functionalized monomers [115,116]. Previously, this strategy has already been successfully applied to improve properties of pyrene-modified probes in [48,90,118].

**Figure 14:**
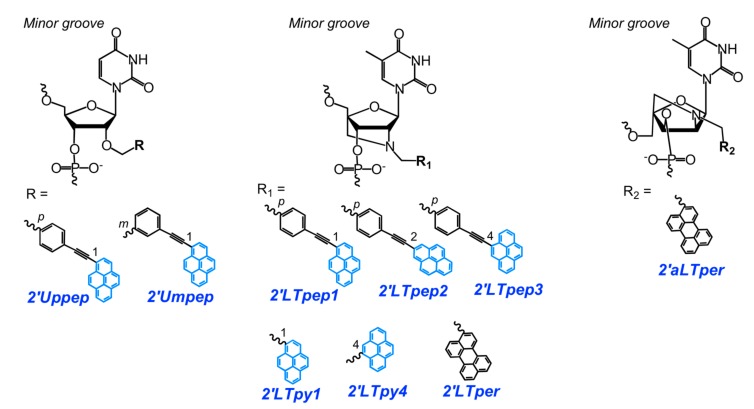
Structures of *O*2′-(phenylethynyl)pyrene-modified RNA, *N*2′-pyrene-modified 2′-amino-LNA, and *N*2′-pyrene-modified 2′-amino-α-l-LNA monomers studied in [14,90,119] and the anticipated disposition of pyrene in the hybrid probe-target duplex.

More recently, Korshun and coauthors reported two new regioisomeric O2′-(phenylethynyl)pyrene-modified monomers, **2′Umpep** (*meta*-) and **2′Uppep** (*para*-) (Figure 14), which were then consecutively incorporated in the center of the DNA or 2′-*O*-methyl RNA probes to form excimer-generating units (four different variants) (Figure 1b) [119]. In some cases, the probes displayed pronounced changes in fluorescence spectra upon binding to fully-matched and mismatched NA targets, namely the 2′-*O*-methyl RNA probe with the **2′Umpep-2′Uppep** (*meta*-*para*-) unit displayed significantly increased excimer emission intensity when hybridized to mismatched RNA compared to a fully-matched RNA target. This allowed three natural SNPs in the *Helicobacter pylori* 23S RNA gene (A2144G, A2143G, A2143C) and the wild-type gene to be distinguished.

Novel *N*2′-(phenylethynyl)pyrene-modified 2′-amino-LNA monomers **2′LTpep1-2′LTpep3** were introduced by Wengel and coauthors for fluorescent-based SNP detection (Figure 14) [90]. Changes in the fluorescence spectra presumably caused by conformational changes of a single, or pair of, DNA/LNA mixed-base probes upon binding to an NA target. Model dual- (Figure 1c) and doubly-labeled (Figure 1b) mixmer DNA/LNA probes comprising monomers **2′LTpep1-2′LTpep3** demonstrated high-affinity hybridization of the probes and excellent fluorescence responses to single-base mismatches in DNA/RNA targets. The authors showed the utility of the probes to characterize the 2332–2354 HIV *pol* fragment with the mutation G2340A causing drug-resistance. The specific fluorescence signal of the probes in a series of 200 clinical samples was observed with low limits of detection and in the presence of additional polymorphisms.

Astakhova and coauthors developed a new assay for the detection of DNA and RNA based on the pyrene-perylene FRET pair attached to short mixmer DNA/LNA probes [14]. They proposed new *N*2′-pyrene-modified 2′-amino-LNA monomer **2′LTpy4**, to vary the geometry of the pyrene fluorophore, and two variants of *N*2′-perylene-modified monomers **2′LTper** (2′-amino-LNA) and **2′aLTper** (2′-amino-α-l-LNA) aiming to increase the efficiency of FRET (Figure 14). Model dual- (Figure 1c) and doubly-labeled (Figure 1b) mixmer DNA/LNA probes comprising different combinations of monomers **2′LTpy1**, **2′LTpy4**, **2′LTper**, and **2′aLTper** were studied with respect to their SNP-discriminating properties. The dual probes demonstrated high sensitivity of fluorescence to single-base mismatches, but not doubly-labeled probes. Using the novel dual probes, SNP detection is achieved with the advantages of a large Stokes shift (115 nm), high fluorescence quantum yields, and a low limit of target detection values (<5–10 nM, of highly-polymorphic HIV *pol* cDNA and RNA) [14].

**Figure 15:**
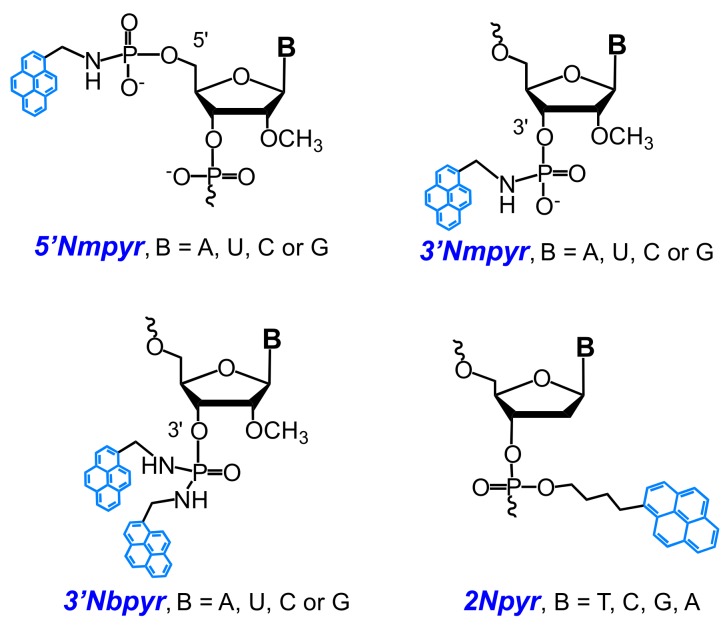
Structures of 5′-mono-(**5′Nmpyr**), 3′-mono-(**3′Nmpyr**), and bispyrene-modified (**3′Nbpyr**) nucleotides [91,120,121], and pyrene-modified monomer (**2Npyr**) [122].

A new detection strategy utilizing highly sensitive and specific fluorescent dual 2′-*O*-methyl RNA probes for the detection of point mutations in DNA was described in [91,120,121]. The detection strategy is based on the use of tandems of 3′- and 5′-mono- and bispyrene-modified 2′-*O*-methyl RNA probes (structures of modified nucleotides are shown in Figure 11 for **5′Nbpyr**, and Figure 15 for **5′Nmpyr**, **3′Nmpyr** and **3′Nbpyr**) forming an excimer when two or more pyrene groups are brought into close proximity upon hybridization of the components of the probes with DNA. The application of the developed probes for detection of C677T polymorphism in the methylenetetrahydrofolate reductase gene (*MHTFR*) has been demonstrated.

New original monomer **2Npyr** with a pyrene moiety attached directly to the internucleotide phosphate was developed and site-specifically incorporated in DNA probes by Tang and coauthors (Figure 15) [122]. The probes displayed a very low fluorescent emission signal due to effective photoinduced electron transfer quenching by nearby nucleobases. They observed a strong fluorescence emission upon binding of the probes to the matched duplex in the case of probes with pyrene modified at the 3′-phosphate of thymidine and cytidine. These new types of pyrene-labeled probes were successfully used as “turn on” sensor for SNP in PCR applications and the *BRAF* single-mutation site of human melanoma. 

**Figure 16:**
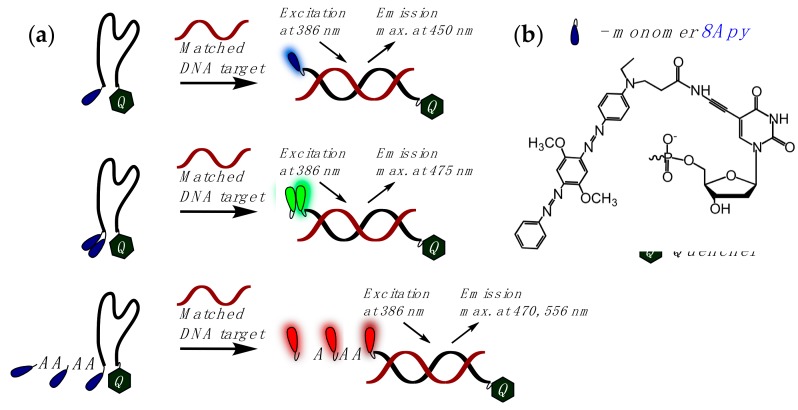
(**a**) Schematic representation of multi color emission folded DNA probing system; (**b**) structure of universal quencher described in [123].

In an interesting SNP typing approach described in [123], one or more insertions of monomer **8Apy** (Figure 13) were incorporated at the 5′-end of a folded DNA probe containing a quencher of fluorescence at the 3′-end (Figure 16). Upon hybridization with the DNA target the probes exhibited a fluorescent signal of three different colors, namely blue, green, and red, based on number of **8Apy** monomers in the probe (one, two, and three, respectively), and could discriminate the fully-matched DNA target from one or two base-mismatched targets.

### 2.3. Fluorescent Biosensors for Detection of Miscellaneous Targets

#### 2.3.1. Fluorescent Aptasensors Containing Pyrene Residues

Nowadays, synthetic fluorescently-labeled NA-aptamers have gained increasing attention as prospective tools in nanobiotechnology, diagnostics, and therapy [124,125,126,127,128,129,130]. Pyrene-labeled aptamers integrated into various biosensing systems are used for the detection of a broad range of biologically-relevant targets, such as proteins, small molecules, and metal ions (IgE, PDGF, lysozyme, thrombin, cocaine, l-argininamide, ATP, GTP, adenosine, potassium ions, and others). In this section, we discuss the most common strategies for the design of aptamer-based detection systems using pyrene as a fluorescent label.

**Figure 17:**
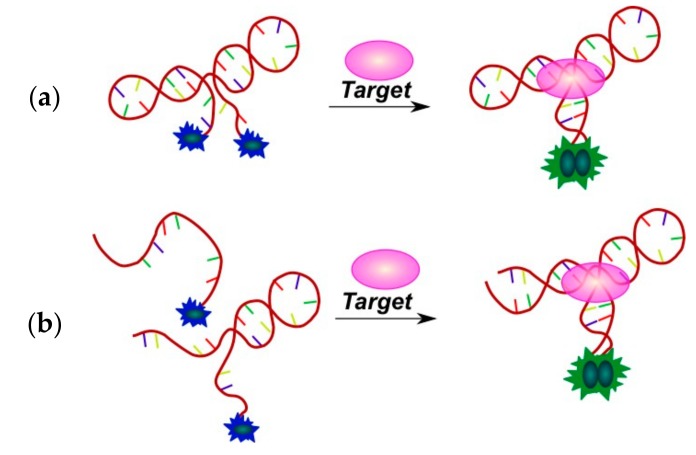
(**a**) Detection of target molecule using a bispyrene-labeled aptamer; (**b**) detection of a target molecule with a two-component aptamer system bearing two pyrene residues.

The first strategy presumes the use of the pyrene moiety directly into the aptamer molecule. Monopyrene-modified aptamers were used for the detection of ATP [97,131] and thrombin [132]. An RNA aptamer conjugated with a pyrene-modified peptide was used for GTP detection in [133]. Very sensitive fluorescent switches were developed by using two pyrene residues attached at the 3′- and 5′-termini of the aptamer (Figure 17a). The binding of a target brings the pyrene moieties of 3′- and 5′-termini of the aptamer stem into close proximity, thus generating an excimer fluorescence signal. This approach was used for the detection of immunoglobulin E (IgE) [134], platelet-derived growth factor (PDGF) [135], and thrombin [136] at the picomolar level in homogeneous solutions. Further development of this approach led to dividing of aptamer molecule into two parts which are associated in a complex upon binding with a target molecule (Figure 17b). A feasibility of this technique was demonstrated by the detection of adenosine [137] and cocaine [137,138].

The bispyrene label can be introduced not only onto aptamer termini, but also into the chain as a non-nucleotidic insertion to yield a fluorescent sensor with a high signal intensity and specificity to a target ligand, e.g., ATP [139,140].

**Figure 18:**
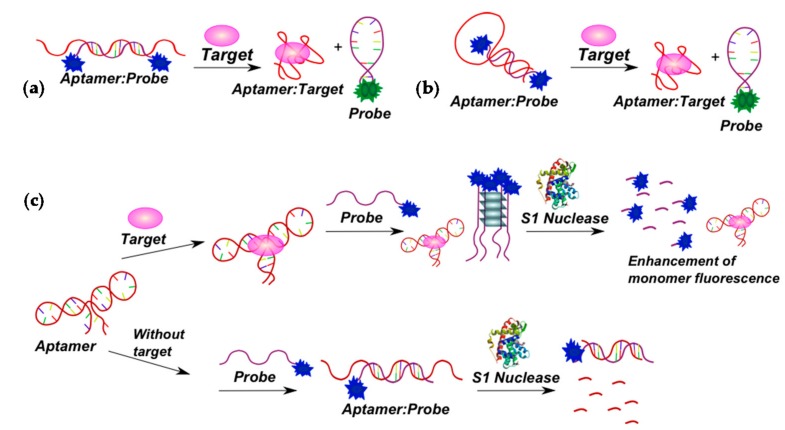
Detection systems using aptamers and signal oligonucleotides (pyrene-containing probe). (**a**) Detection of a target with a bispyrene-modified hairpin DNA probe partially complementary to an aptamer; (**b**) detection of a target using a triple-helix molecular switch (THMS) system; and (**c**) detection of a target with a multi-component system containing a monopyrene-modified probe, an aptamer, and S1 nuclease.

In the second strategy, sensor systems consist of an aptamer and a pyrene-modified signal oligonucleotide. Various types of these systems are presented in Figure 18. In the absence of a target, an aptamer and a bispyrene probe (signal oligonucleotide) form a complex and both pyrene moieties are spatially separated. Aptamer-target binding results in the formation of the structured complex, thus liberating the pyrene excimer-forming probe (Figure 18a,b). The aptamer-probe complex can be either an oligonucleotide duplex [141] or a triplex (triple-helix molecular switch (THMS) system) [142]. These systems were applied for the detection of lysozyme [141], thrombin [142], ATP [142], and l-argininamide [142].

Immunoglobulin E is considered to be an attractive target for aptamer selection and design of aptamer-based detection assays [126]. A multi-component system constructed for the IgE detection was reported in [143] (Figure 18c). After incubation of the anti-IgE aptamer with the target, a monopyrene-modified G-rich probe complementary to the aptamer sequence and able to form an intermolecular parallel tetrameric G-quadruplex was added. The probe forms a duplex with the free aptamer, but not with the target-bound one. Unbound probe molecules form a quadruplex, and after specific S1 nuclease DNA digestion, pyrene is liberated, so monomer fluorescence arises. 

**Figure 19:**
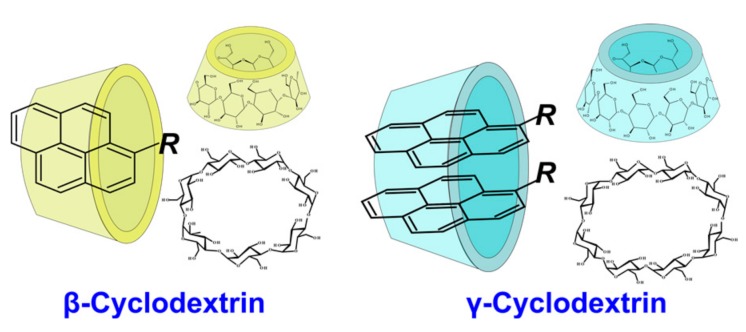
Host-guest complexes between pyrenes and cyclodextrins (β-cyclodextrin (β-CD), and γ-cyclodextrin (γ-CD)).

The third strategy implies the use of cyclodextrin to enhance the pyrene fluorescence [144,145,146,147]. Extensive studies have documented that the truncated, corn-shaped hydrophobic cavity of cyclodextrin (CD) can selectively bind diverse organic molecules to form a versatile supramolecular assembly (Figure 19). 

**Figure 20:**
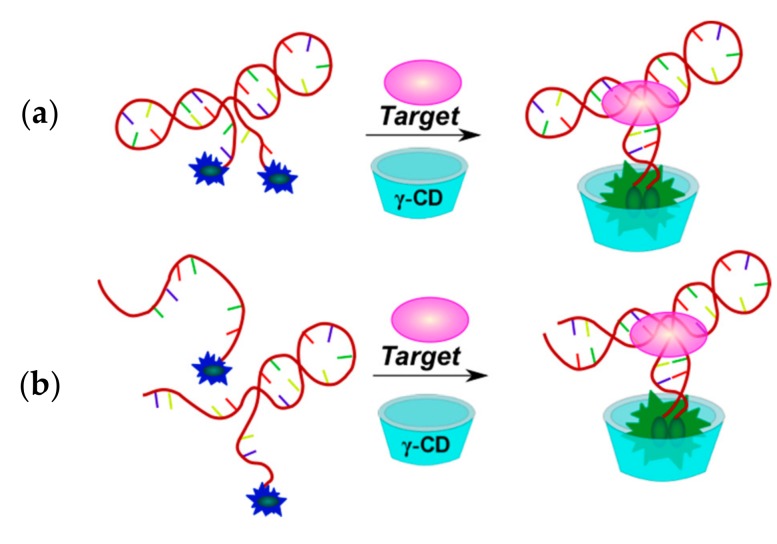
Detection of molecular targets using a bispyrene-labeled aptamer [147] (**a**); a two-component aptamer [145] (**b**); and γ-CD.

A γ-CD-mediated dual pyrene-labeled stemless molecular beacon (γ-CD-P-MB) was constructed by bounding the bispyrene labeled DNA probe in the γ-CD hydrophobic cavity [147]. By introducing γ-CD amplification, the two pyrene molecules are hosted within the γ-CD cavity to trigger remarkable excimer fluorescence enhancement. γ-CD/pyrene bounding interaction also allows tuning the stem stability of the probes, thereby achieving higher target-binding selectivity and sensitivity than conventionally-structured DNA probes. The γ-CD-P-MB avoids any variation of the stem length and sequences, which eliminates restrictions on probe design. In addition, the Stokes shift (about 130 nm) and long fluorescence lifetime (about 40 ns) of the pyrene excimer (λ_ex_ = 344 nm, λ_em_ = 475 nm) prevents background interference, such as autofluorescence from biological environments, thus providing an opportunity for the detection of targets from complex biological media. Such systems were applied for DNA detection [147,148] and SNP [95]. Bispyrene-labeled aptamers were used for thrombin [147] and ATP [145] detection in the presence of γ-CD (Figure 20).

**Figure 21:**
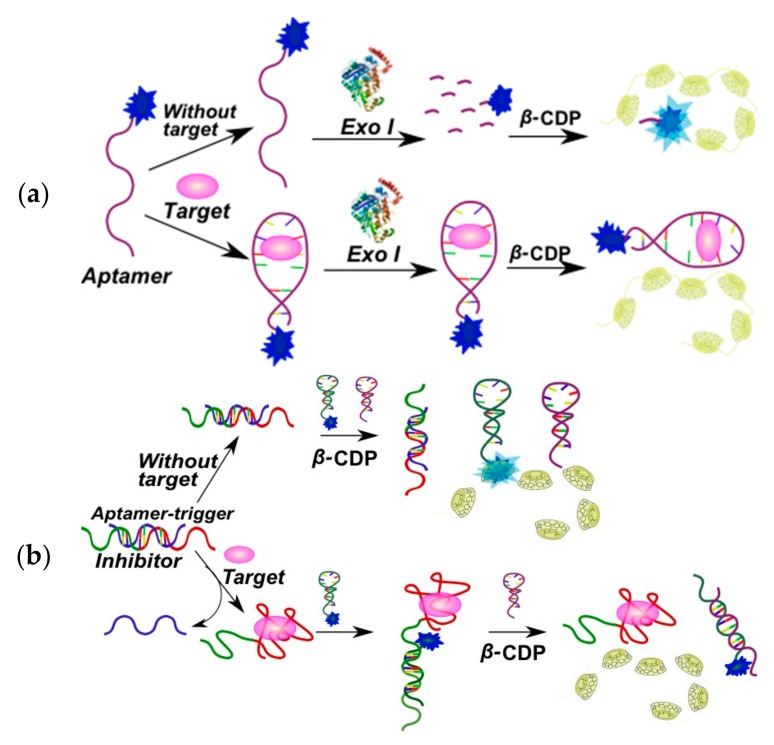
(**a**) System for adenosine detection using pyrene-modified aptamer, β-cyclodextrin polymer (β-CDP) and Exo I [146]; (**b**) System for adenosine detection using a pyrene-modified hairpin probe, the complementary non-fluorescent probe, β-CDP, inhibitor, and aptamer-trigger molecule [144].

Several fluorescence analytical methods based on the host-guest interaction between the epichlorohydrin cross-linked β-cyclodextrin polymer (β-CDP) and pyrene were developed [149]. Compared to the monomer β-CD, β-CDPs are more soluble in water and can achieve better pyrene fluorescence enhancement. The signal amplification is based on host-guest interactions between β-CDP and pyrene, and does not require a quencher. That is why it can solve the issue of complex probe design and also provide further sensitivity improvements. These systems were used for DNA [111,146,150] and miRNA [149,151] detection. Multi-component systems for the detection of adenosine on the basis of aptamers and β-CDP were reported [144,146] (Figure 21).

An adenosine-specific monopyrene-labeled aptamer (Py-aptamer) has been used as an aptasensor component (Figure 21a) [146]. Binding of the adenosine with the aptamer results in the formation of a stable double-stranded structure at the terminus of the Py-aptamer, which inhibits the hydrolytic activity of Exo I nuclease.

A more sophisticated detection scheme was reported in [144] (Figure 21b). The system includes two hairpin probes and an aptamer-trigger/inhibitor duplex probe, and the pyrene-labeled probe as the signal unit. In the absence of adenosine, the aptamer-trigger was inhibited by the inhibitor strand. The probes were in the hairpin conformation without activation of the trigger strand. The pyrene residue at the 5′-terminus of the single-stranded stem of the labeled probe could be easily trapped in the hydrophobic cavity of β-CDP because of weak steric hindrance, which leads to a significant fluorescence enhancement. Once the hairpin assembly was catalyzed by the adenosine-aptamer binding event, trigger part of aptamer-trigger molecule formed a duplex with the pyrene-labeled probe and a hybridized duplex of two hairpin probes was created continuously. The pyrene label at the 5′-terminus of the duplex of two hairpin probes can hardly enter the cavity of β-CDP due to the steric hindrance, thus giving a weaker fluorescence signal. Therefore, the target could be detected by this simple mix-and-detect amplification method.

The detection schemes based on the host-guest interaction of CD-based and pyrene-labeled probes might be feasible for detecting a wide range of target species with high sensitivity and selectivity.

#### 2.3.2. Pyrene-Labeled Oligonucleotide Constructions for the Detection of Metal Ions (K^+^, Hg^2+^, Zr^4+^, Cu^2+^)

**Figure 22:**
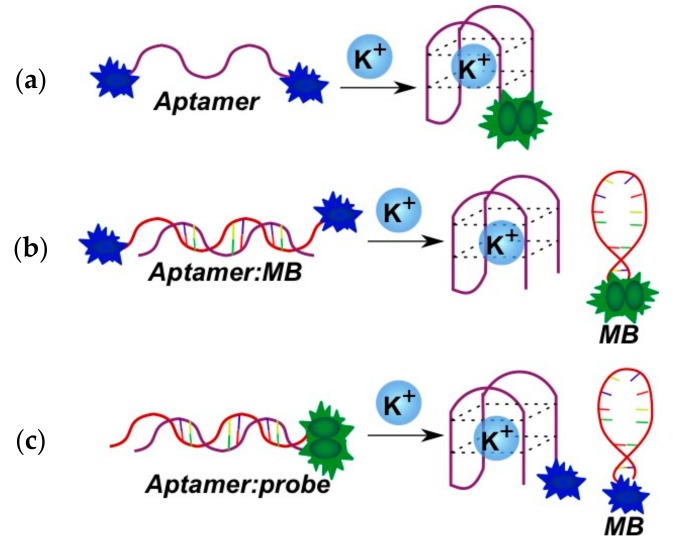
Aptamer-based systems for potassium ion detection. (**a**) Bispyrene-labeled aptamer; (**b**) bispyrene-labeled probe (molecular beacon); and (**c**) monopyrene-labeled aptamer and probe.

The thrombin aptamer is widely used for potassium ion detection (K^+^) due to its ability to form a quadruplex in the presence of these ions. Three variants of fluorescent systems for K^+^ detection were designed with the use of a pyrene as a fluorophore. Pyrene residues were introduced into an aptamer [152], a signal oligonucleotide (bispyrene-labeled molecular beacon) [153] or into both components of the detection system [154] (Figure 22). The detection is accomplished by the registration of either quenching [154] (Figure 22c) or enhancement of the excimer fluorescence [152,153] (Figure 22a,b).

**Figure 23:**
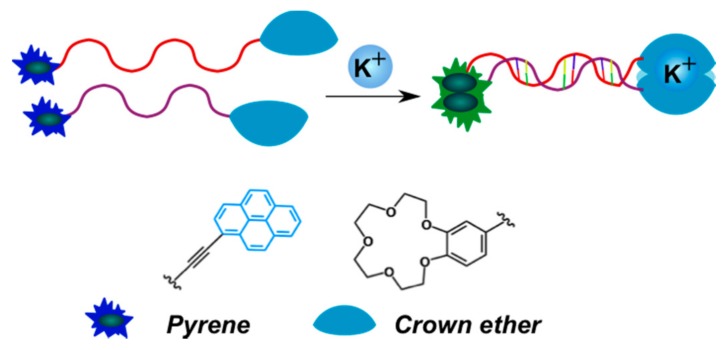
Schematic representation of the duplex-based potassium sensor with monomer-excimer switching [155].

Another system for the detection of potassium ions is built of two complementary monopyrene-labeled oligonucleotides that exhibit pyrene excimer fluorescence when the duplex is assembled [155]. Two crown ether moieties are at the opposite termini of the duplex, binding potassium ions (Figure 23). This duplex is stable only in the presence of potassium ions, which permits their detection by the excimer fluorescence signal.

**Figure 24:**
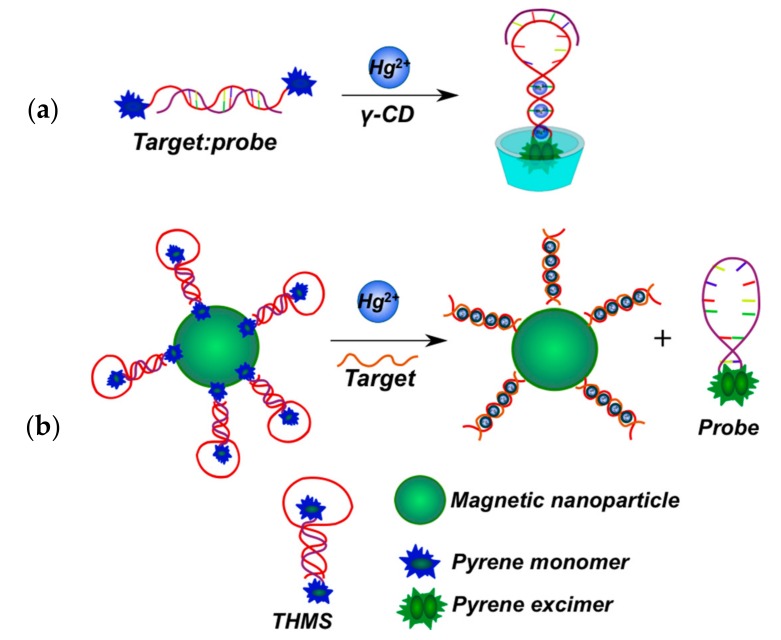
(**a**) Sensing principle for Hg^2+^-detection using γ-CD [157]; (**b**) THMS for Hg^2+^-detection [158].

Methods of mercury(II) ion detection are based on thymine coordination [156]. Hg^2+^ ions can specifically coordinate T-T pairs to form T-Hg^2+^-T complexes. In the first approach, a bispyrene-labeled molecular beacon able to form a hairpin in the presence of Hg^2+^ ions was used as a sensing element for the detection system [145] (Figure 24a). γ-CD addition increased the excimer fluorescence. The pyrene dimers can be formed owing to the cooperative interactions of the T-Hg^2+^-T base pairs and the inclusion into γ-CD, resulting in an increase of pyrene excimer fluorescence. In the presence of γ-CD the response of the probe system to Hg^2+^ is quite selective.

A thymine-rich mercury-specific oligonucleotide (MSO) acted as the capture probe in [158] (Figure 24b). A bispyrene-labeled oligonucleotide (molecular beacon) was used as a signal probe. To eliminate the quenching of Hg^2+^-induced pyrene excimer fluorescence, magnetic nanoparticles conjugated with MSO were first hybridized with a signal probe to form a hairpin-shaped triple-helix molecular switch (THMS) through Watson-Crick and Hoogsteen base pairing. Binding of Hg^2+^ with the MSO releases the signal probe, leading to pyrene excimer emission as a result of the conformation rearrangement of the probe. 

**Figure 25:**
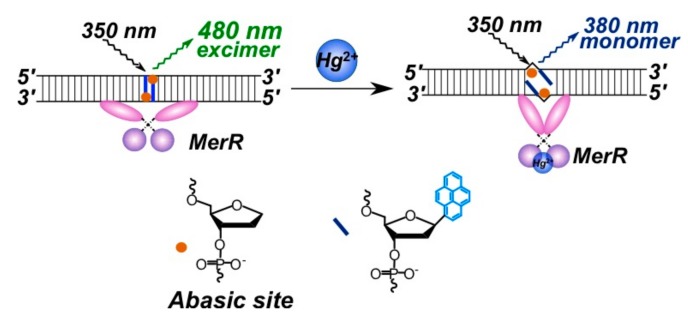
Sensing principle for Hg^2+^ detection using MerR protein [159].

The third approach implies the use of MerR protein for mercury detection [159]. MerR protein recognizes its specific target metal ion and causes a distortion of the bound duplex DNA. Specifically, the H-bonds in the middle of the sequence are disturbed. The authors used two complementary 31-mer oligonucleotides containing the specific MerR binding sequence, each containing one pyrene in the center of the sequence opposing an abasic site to ensure base stacking of pyrenes in DNA (Figure 25).

**Figure 26:**
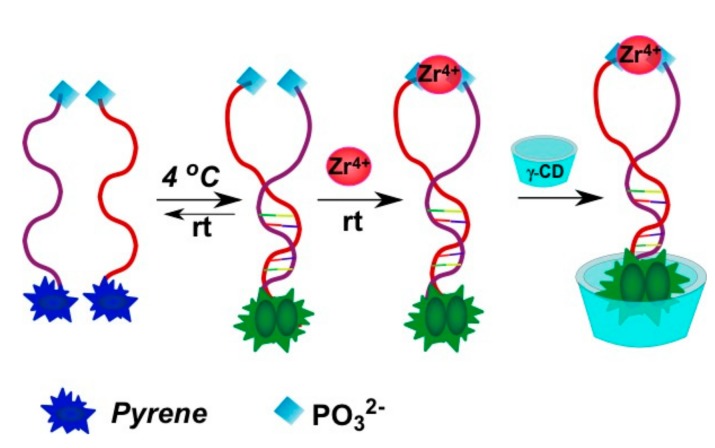
A schematic presentation of molecular beacon-based sensing system for the amplification detection of Zr^4+^ using γ-CD as a signal amplifier [93].

An interesting strategy was developed for Zr^4+^ ion detection [93]. Zr^4+^ ions could selectively coordinate two phosphate-functionalized molecules. Zr^4+^-sensitive probe undergoing the target-triggered hairpin structure formation was designed. In the molecular beacon-based sensing system, two phosphorylated and pyrene-labeled oligonucleotides represent both recognition and reporter units, which were designed to complement each other at the pyrene-labeled termini and, therefore, could form a DNA molecular beacon upon the interaction with Zr^4+^ (Figure 26). The γ-CD was used as an amplifier of the excimer fluorescence.

**Figure 27:**
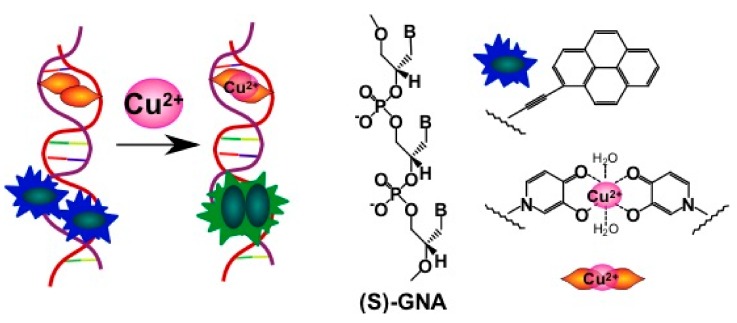
Design of a GNA-based Cu^2+^ sensor [160].

A sensor made of two complementary chains of GNA (glycol nucleic acid) bearing the pyrene acetylides and hydroxypyridones was designed for Cu^2+^ ion detection [160]. The effect of metal-mediated hydroxypyridone homo-base pair formation results in the stabilization of the complementary complex and in the pyrene excimer formation (Figure 27).

In the absence of Cu^2+^ ions, the duplex is not stable, and formation of the excimer is impossible. Dose-dependent fluorescence was registered for Cu^2+^ ions. Pyrene acetylide GNA nucleotides enable the monitoring of duplex formation by excimer fluorescence detection, which was successfully applied to the design of a fluorescent copper(II) ion sensor.

#### 2.3.3. Pyrene-Labeled Oligonucleotide Constructions for Detection of Other Small Molecules

**Figure 28:**
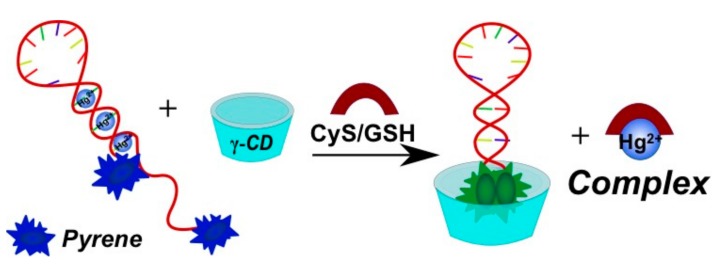
A schematic description of sensing scheme based on cyclodextrin (CD) supramolecular inclusion-enhanced pyrene excimer switching [162].

Small molecules can be detected not only by means of specific aptamer molecules, but also using other types of specific interactions. Oligonucleotide constructions bearing pyrene residues as fluorophores were used for the detection of porphyrins [161], fatty acids, and biothiols (for instance, sulfur-containing amino acids) [162]. Systems analogous to the one proposed in [155] were designed in [161,163] (Figure 28). Cyclodextrin molecules were used as sensing elements for porphyrins and fatty acids. These hydrophobic guest molecules are recognized by two CD residues, which lead to the stabilization of the duplex and pyrene excimer formation, resulting in excimer fluorescence being used as a detection signal.

An efficient pyrene excimer signaling-based fluorescent sensor on the basis of thymine-Hg^2+^-thymine coordination was reported in [162] for the measurement of biothiols (cysteine (CyS), homocysteine (Hcy), glutathione (GSH)) in human serum (Figure 28). In the presence of thiol-containing amino acids or peptides, these molecules bound Hg^2+^ and displace it from the T-Hg^2+^-T complex, thereby distorting the T-Hg^2+^-T structure. The γ-CD induced the pyrene excimer fluorescence enhancement.

#### 2.3.4. Study of Enzymes and DNAzymes Activity Using Pyrene-Labeled Oligonucleotides

An extensively developing field of application of pyrene-modified oligonucleotides is the design of systems for the investigation of enzymes and DNAzyme activities. Two strategies for T4 polynucleotide kinase activity detection were developed, implying a gamma-cyclodextrin (γ-CD) [164] or β-cyclodextrin polymer (β-CDP) [165] and an exonuclease reaction. An analogous strategy was used for alkaline phosphatase (ALP) activity detection [150]. One or two monopyrene-modified oligonucleotides acted as sensing components of the detection systems. The pyrene moiety was attached to the 5′- or 3′-termini of oligodeoxyribonucleotides. 

**Figure 29:**
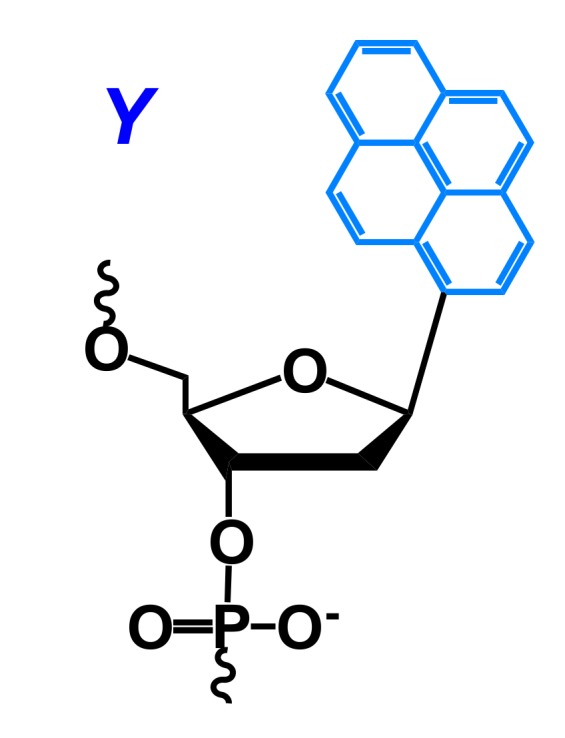
The fluorescent monomer (**Y**) used in [166,167,168,169,170].

The introduction of non-natural fluorescent monomer **Y** (Figure 29) into the oligonucleotide substrate of different enzyme bases was introduced by Kool and colleagues. These modified substrates were used to study the activity of a human oxidative enzyme (ALKBH3) [166], an 8-oxoguanine glycosylase 1 (OGG1) [167], a 3′-exonuclease (ExoT, RNAse T), a 5′-exonuclease (single-stranded-DNA-specific 5′-exonuclease RecJf), a single-strand endonuclease (nuclease S1) [168], and bacterial [169,170] and mammalian uracil DNA glycosylase (UDG) [170]. 

**Figure 30:**
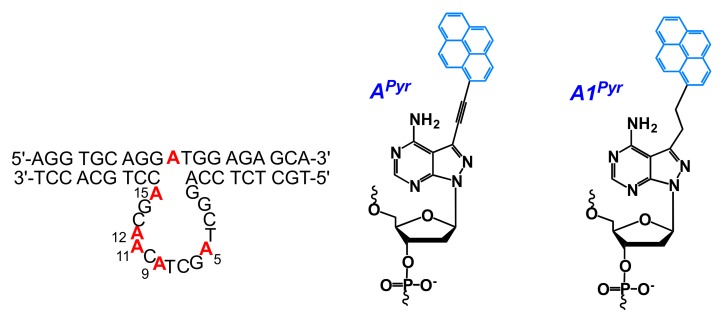
DNAzyme-substrate complexes modified with a combination of two pyrene-modified adenosines (**A^Pyr^** or **A1^Pyr^**) in the substrate and in the DNAzyme catalytic core.

The system of pyrene-modified substrate and pyrene-modified DNAzymes was designed for the conformational study of the DNAzyme-substrate complex in solution [171]. Two fluorescent nucleoside analogues **A^Pyr^** or **A1^Pyr^** enabled the introduction of a pyrenyl group at one of the five dA residues in the catalytic core and the unpaired adenosine residue in its full-DNA substrate, respectively (Figure 30). The position-dependent quenching effect of pyrene emission fluorescence by nucleobases, especially the pyrene-pyrene interaction, was observed for some positions. Catalytically-relevant nucleobases were identified for understanding the catalytic mechanism of 10–23 DNAzyme.

#### 2.3.5. Monitoring Formation of G-Quadruplexes and i-Motifs 

Single-stranded guanine-rich DNA sequences can form four-stranded helical DNA structures (called G-quadruplexes) through a cyclic Hoogsten hydrogen-bonding arrangement of four guanines with each other. G-quadruplex formation is driven by monovalent cations such as Na^+^ and K^+^, and hence physiological buffer conditions favour their formation [172]. G-quadruplexes are prospective tools providing the researcher with the means of manipulating DNA/RNA assemblies in ways not possible using standard nucleotides [35]. Specific fluorescence properties of pyrene, e.g., an ability to form excimers and a large number of techniques available for its detection, contributed to a great interest in applying pyrene-modified oligonucleotides for G-quadruplexes studies [173,174,175,176,177,178].

**Figure 31:**
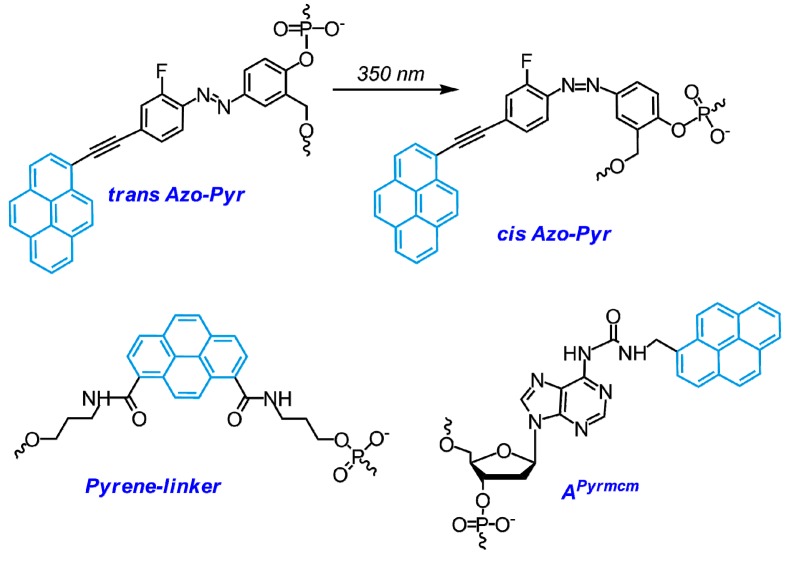
Structures of pyrene-modified nucleotide (**A^Pyrmcm^**) and non-nucleotidic pyrene linker (**Pyrene-linker** and **Azo-Pyr**) employed for the investigation of G-quadruplexes.

Pyrene moiety was introduced into oligonucleotides using modified adenosine (**8Apy**, **A^Pyrmcm^**) [173,178,179], modified uridine (**5Upy**) (Figure 12) [177,180] or non-nucleotidic monomer (**Pyrene-linker**, **Azo-Pyr**) [175,176,181] (Figure 13 and Figure 31).

**Figure 32:**
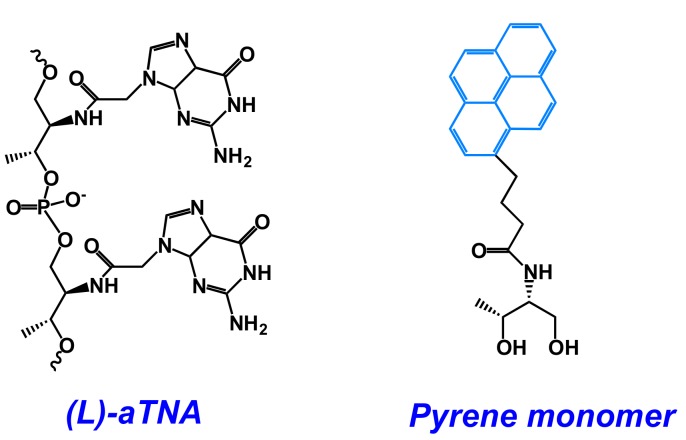
Structures of acyclic (l)-threoninol nucleic acid (**(L)-aTNA**) and pyrene residue on a TNA scaffold (**Pyrene monomer**) [175].

In [175], pyrene-modified oligonucleotides were applied for structural studies of non-natural G-quadruplex structures made of acyclic (L)-threoninol nucleic acid (**(L)-aTNA**) (Figure 32).

The unique tetrameric assembly of a DNA/RNA duplex bearing a pyrene-modified deoxyadenosine residue at the 5′-end of DNA and a 3′-GAGGG sequence at the 3′-end of the RNA to form the tetra-duplex complex was studied in [178]. A simple and sensitive system for detecting RNA AGG trinucleotide repeats through the formation of intermolecular G-quadruplexes using a fluorescent oligonucleotide was developed [177].

The above-mentioned examples demonstrate that pyrene-modified oligonucleotides represent unique instruments for the study of different G-quadruplex structures.

Cytosine-rich DNA strand can induce i-motifs under acidic conditions, i.e., two parallel-type duplexes with intercalating protonated cytosine-cytosine (CH^+^·C) pairs in an antiparallel orientation. These compact structures are often found in human telomeric DNA and regulatory regions of eukaryotic chromosomes [182], therefore the probing of i-motif structures is of particular importance. Several fluorescent probes for sensing of i-motif transitions based on pyrene-modified oligonucleotides have been proposed [92,110,183,184,185,186,187,188,189,190].

Kim and coauthors have suggested a series of novel quencher-free fluorescent probes that are able to distinguish the conformational change from single-stranded, duplex, and i-motif structures [184,185,186]. The probes are C-rich oligonucleotides modified by one or two insertions of monomer **8Apy** (Figure 13), that is known to be very sensitive to its local environment [191]. In [184], the monomer **8Apy** was attached to the 3′-end of a 23-mer sequence composed of repetitive arrays of the eukaryotic telomeric strand. The single-stranded 23-mer (at pH 7.2) exhibits strong emission band at λ_max_ 452 nm. Upon formation of i-motif structure (at pH 4.0), the fluorescence intensity of the probe decreases by ~1.8-fold due to end-stacking of pyrene moiety with nucleobases. Addition of a complementary G-rich strand results in quenching of fluorescence of the probe. Then, a fluorescent system based on stable stacking interactions of the two nonpolar monomers **8Apy** inserted in terminal and mid-loop positions of C-rich DNA has been developed for monitoring i-motif transitions through exciplex emission [185]. Later, two alternative multicolor exciplex-forming systems have been reported for probing of the structural dynamics of i-motif of the retinoblastoma gene fragment [186,187].

Dembska and Juskowiak have reported the dual pyrene-functionalized molecular beacons as pH-sensitive fluorescent probes [92,110]. These probes consist of a C-rich pH-sensitive domain (C_4_GC_4_GC_4_GC_4_TA) in their loops. These molecular beacons exhibit excimer fluorescence (480 nm) at acidic pH, whereas increasing pH causes i-motif unfolding, followed by the stem destabilization and by the pyrene separation (emission at ~400 nm) [92]. The feasibility of such fluorescent pH-sensitive probes was examined by determining the linear response range and reversibility of fluorescence response to pH cycling. Two probes were delivered into HeLa cells and their suitability for pH change sensing in living cells was demonstrated [110]. 

Choi et al. have shown that the effective photoinduced electron transfer occurs in i-motif structure of the human telomeric DNA fragment comprising monomer **5Upy** (Figure 12) and anthraquinone (donor and acceptor, respectively) [189]. The findings confirm that i-motif structures can be considered as promising platforms for the development of novel nanoelectronic devices.

#### 2.3.6. Nucleic Acid-Based Polyfluorophores Comprising Pyrene Derivatives

**Figure 33:**
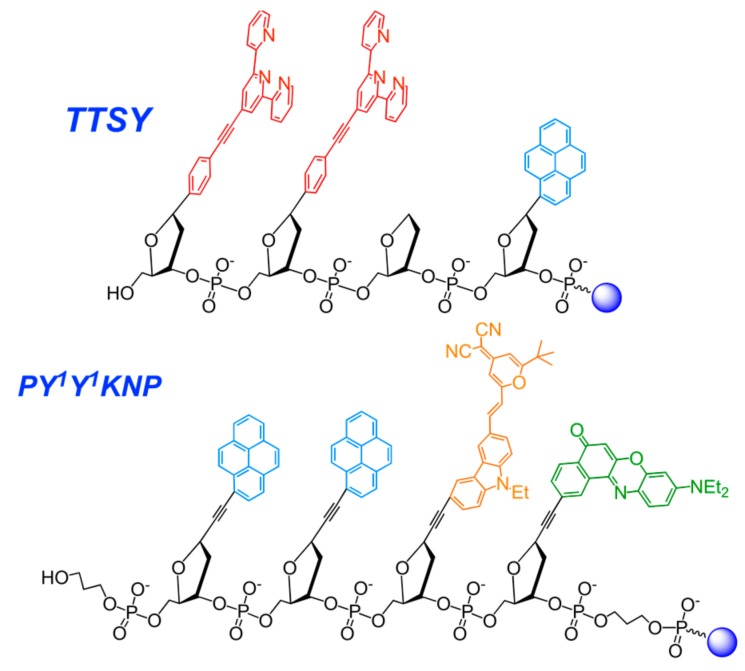
The examples of the polyfluorophores (**TTSY** and **PY^1^Y^1^KNP**) covalently attached to polyethylene glycol-polystyrene bead. Et: ethyl.

Kool’s group have suggested a new approach for discovering useful new classes of fluorescent tags [192,193] (Figure 33) based on the concept of polyfluorophores assembled on a DNA backbone. They have designed oligonucleotide analogues, where fluorescent chromophores are incorporated into single large DNA-like molecules in such a manner that they can interact closely with one another. This close association resulted in interesting and sometimes surprising changes in their fluorescence behavior (e.g., emission color and intensity), and in the ability to selectively detect molecules. The combinatorial chemical libraries on polymer beads were built and useful reporter molecules were selected by fluorescence microscopy [194,195,196,197].

Screening of the library of 1296 tetrameric compounds immobilized on polystyrene microbeads revealed a set of chemosensor sequences that respond strongly to a series of structurally-varied pesticide analytes. A set of ten chemosensors on microbeads enables discriminating of fourteen different pesticides of several structural classes [195]. A small set of chemosensors built on a DNA backbone generated from this library was able to readily discriminate seventeen anion pollutants in aqueous media at micromolar concentrations [194]. Thus, the pyrene-modified oligonucleotide constructions have been successfully used for the design of diverse sensing systems for the detection of small molecules, metal ions, and various biomolecules.

Another example of π-stacked oligonucleotide-based polyfluorophore arrays was reported by Wagenknecht’s group [198]. 5′-Fullerene-conjugated homo-oligonucleotide was used as a template for the polyfluorophore stacks of nucleosides bearing pyrene and Nile red dyes as nucleobase modifications. Being organized via complementary interactions with the template, this supramolecular assembly possesses an electron transfer between dyes and fullerene moieties, with almost the whole UV-VIS absorption spectrum being covered. This associate was used as a light-harvesting block in indium-tin oxide solar cells. Alternatively, electron transfer between fluorescent dyes was achieved by incorporating dye-modified nucleotides into an oligonucleotide using conventional synthesis [199].

Recently, Nakamura et al. constructed a pyrene π-stack array on RNA, which consists of up to ten pyrene moieties and extends to about one helical pitch along the minor groove of RNA or 2′-*O*-methyl RNA duplex, with the use of pyrene-modified **OMUpy** and **OMApy** pairs assembled as “0 interstrand zipper arrangements” (Figure 2b) [46,51,52,53]. The duplexes with the pyrene zipper arrays display high thermal stability and exhibit strong static excimer fluorescence due to direct excitation of associated pyrenes in the ground state, indicating great potential advantages in applications to fluorescent probes and electronic devices.

## 3. Agents for Targeting of dsDNAs

Development of agents for sequence-specific targeting of dsDNAs is an actual task of molecular biology and medicine, as the probes could become valuable tools for the regulation of the gene expression via inhibition of transcription, correction of mutations in DNA, and visualization of chromosomal DNA. To the moment, several promising approaches based on the use of simple agents as triplex-forming oligonucleotides (TFOs) [200,201,202], dsDNA Invader probes [17], PNAs [203], pseudocomplementary PNA [204], and minor groove-binding polyamides [205] as sequence-specific dsDNA binding probes have been suggested. Along with them, more sophisticated agents for dsDNA targeting, i.e., DNA-recognizing engineered peptides such as “zinc finger” nucleases based on DNA-binding domains of eukaryotic transcription factors, transcription activator-like effector nucleases (TALENs), and CRISPR/Cas9 systems are currently of special interest [206,207,208].

Pyrene-modified oligonucleotides have been extensively explored in the frame of two different approaches for dsDNA targeting, namely, (1) through the formation of stable triplexes using TFOs comprising TINA (Twisted Intercalating Nucleic Acids) monomers; and (2) through the partial DNA duplex unwinding and isosequential binding by Invader probes, i.e., short DNA duplexes modified with one or more +1 interstrand zipper arrangements of intercalator-modified nucleotides. In both methods, the ability of the pyrene moiety to intercalate into the DNA duplex core and previous findings on structure-based binding modes of pyrene moieties in NA duplexes (generalized in review [4]) were used for the rational design of the probes.

### 3.1. TINA-Modified Triplex Forming Oligonucleotides

**Figure 34:**
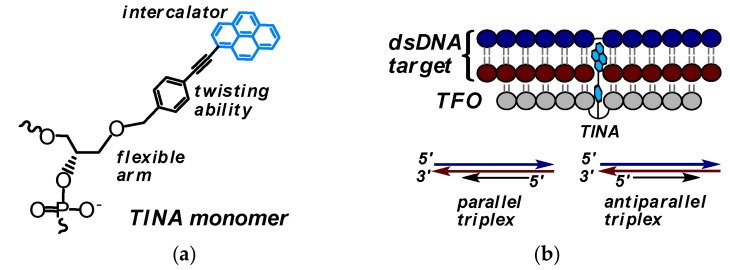
(**a**) Chemical structure of the **TINA** monomer; (**b**) Schematic representations of the triplex formed upon binding of TINA-TFO to dsDNA target (upper) and strand orientation in triplexes (lower).

In the frame of the first approach, Pedersen, Filichev and co-workers have suggested an original class of TFOs comprising insertions of (*R*)-1-*O*-[4-(1-pyrenylethynyl)phenylmethyl]glycerol monomer (**TINA** monomer) (Figure 34a) prepared by elegant post-synthetic strategy with the use of Sonogashira solid-phase coupling reactions [16]. **TINA** monomers were designed as bulge insertions in the TFOs (TINA-TFOs) with the intercalating pyrene moiety attached to a linker consisting phenyl ring through a triple bond (Figure 34a). 

Upon triplex formation, the pyrene moiety is positioned inside the DNA duplex between base pairs, and the phenyl ring stacks with nucleobases of the TFO (Figure 34b) (confirmed by molecular modeling studies [209,210]). The authors showed that TINA-modified CT-rich TFOs can form extraordinary thermally-stable Hoogsteen-type (parallel, Figure 34b) triplexes even at neutral pH (in the case of two insertions of **TINA** *T*_m_ was 43 °C at pH 7.2) [16]. In addition, extraordinary stabilization of a DNA three-way junction (Δ*T*_m_ was up to 15.5 °C at pH 7.0) was observed upon insertion of the TINA monomers in the junction region as a bulge [211].

To expand the range of possible binding sites, the alternate-strand TINA-TFOs consisting of a pair of TFOs with inverted polarity and linked to each other through an intercalating 5′-5′-linker have been designed [212]. Later, the specific design rules for positioning of **TINA** monomers in Hoogsteen-type TFOs were determined with respect to their binding properties [213]. G-rich TFOs comprising bulge insertions of **TINA** monomers in the middle of the strands have been demonstrated to display a low tendency of potassium-induced self-aggregation and to form selectively antiparallel triplexes (see the scheme in Figure 34b) that are even more stable than parallel triplexes [210,214]. The findings confirm that the TINA-TFOs have a great potential as sequence-specific tools for genome targeting in vivo. More recently, in order to improve the specificity and to facilitate cellular penetration, the high affine TINA-TFOs (antiparallel and parallel types) have been conjugated to pyrrole-imidazole polyamide minor groove binders (MGB). However, it was shown that the MGBs, as part of the conjugates, do not affect the binding properties of the parent TINA-TFOs [215].

**Figure 35:**
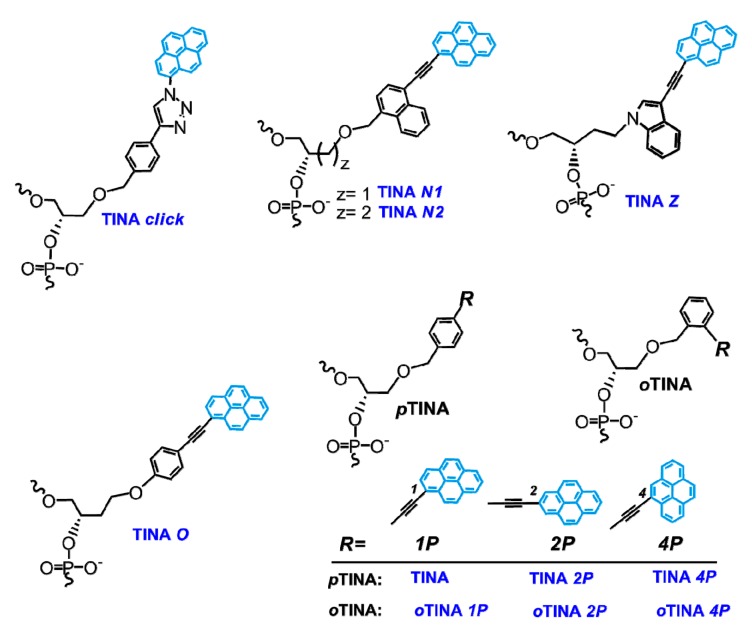
Structures of TINA monomers.

At the same time, several studies have been devoted to the selection of alternative **TINA** monomers that would possess improved stabilizing properties. All the proposed candidates have similar structure with moderate variations in length and structure of linkers and type of intercalators (see, for instance, Figure 35) [209,216,217,218]. For instance, the authors of [217] have developed TINA-TFOs possessing bulged insertions of (*R*)-3-*O*-{4-[1-(pyren-1-yl)-1H-1,2,3-triazol-4-yl]benzyl}glycerol (Figure 35, monomer **TINA *click***) obtained by the post-synthetic labeling with the use of microwave-accelerated [2 + 3]-cycloaddition reaction. These TINA-TFOs form parallel triplexes with high thermal stabilities (up to 40.0 °C at pH 7.2 in the case of three insertions separated by three nucleobases).

Then, a series of novel TINA monomers containing 1-, 2-, or 4-ethynylpyrene residues at the *para*- or *ortho*- positions of (*R*)-1-*O*-phenylmethylglycerol (***p*TINA** and ***o*TINA**, respectively, Figure 35) has been introduced and studied with respect to the thermal stability of the parallel triplexes and fluorescent properties compared to the properties of initial INA and TINA molecules [216]. The insertions of ***p*TINA** were found to be more efficient for Hoogsteen-type triplexes and duplexes, whereas ***o*TINA** analogues can stabilize both Hoogsteen- and Watson-Crick-type complexes. The authors of [209] have reported an optimization of the structure of the TINA monomer based on a molecular modelling examination of its intercalating properties. They investigated the influence of size of aromatic linker and length of a flexible glycerol linker (Figure 35, monomers **TINA *N1*** and **TINA *N2***) and ether position in the glycerol linker (Figure 35, monomer **TINA *O***) of TINA monomers on thermal stability of parallel triplexes formed by the TINA-TFOs with dsDNA targets. They showed that increase of the aromatic surface of linker in case of (*R*)-1-*O*-[4-(1-pyrenylethynyl)naphthylmethyl]glycerol (**TINA *N1***) where the benzene ring has been substituted by naphthalene and the glycerol linker has an optimal length, leads to enhancement of π-π-interactions with nucleobases of TFO and promotes additional thermal stabilization (Δ*T*_m_ = +2.0 °C at pH 7.2 compared to original **TINA** monomer). No significant importance of ether position was found [209]. More recently, the authors of [218] have expanded diversity of TINA monomers introducing novel monomer **TINA *Z*** (Figure 35). They assumed that the replacing of benzene ring with indole would change spatial arrangement of pyrene moiety, which may lead to improving of binding ability of the TINA-TFOs. Actually, the TINA-TFOs comprising monomer **TINA *Z*** show high thermal stability of parallel triplexes at pH 6.0, 6.5, and 7.2 (Δ*T*_m_ compared to unmodified triplexes were +15, +19 and >17 °C, respectively). However, potential applications of the monomer **TINA *Z*** are limited by the formation of side products due to easy hydration of the triple bond under acidic conditions during the synthesis of corresponding amidite or the DNA synthesis.

Due to the remarkable ability of TINA-insertions to stabilize NA complexes through intercalation, various TINA-modified oligonucleotides have been further studied as dsDNA targeting fluorescence probes [216,219,220], ssDNA targeting fluorescent probes with increased sensitivity and specificity [221], improved primers for PCR assays [222], G-quadruplex-forming oligonucleotides [223,224,225,226,227,228], i-motif-forming oligonucleotides [229], aptamers with improved activity [230], probes for DNA duplex invasion [231], and antisense oligonucleotides for splice modulation by induced exon-skipping in vitro [232]. Interestingly, modification of a template oligonucleotide by 5′-terminal ***o*TINA** was shown to reduce or even inhibit of a non-templated nucleotide addition activity of DNA polymerases [233]. Moreover, TINA monomers possess interesting photochemical properties. For example, TINA monomers being inserted at terminal position of DNA duplexes can form supramolecular complexes with cationic porphyrin. Formation of these complexes results in TINA fluorescence quenching due to ground state complex formation [234]. More recently, the authors of [228,235] have shown that TINA monomers attached at the terminal positions of DNA complexes (duplexes or G-quadruplexes) can act as acceptor of energy in photochemical upconvertion.

### 3.2. Invader Probes

An alternative concept for dsDNA targeting was proposed by Hrdlicka and co-authors [17] and by Pedersen and co-authors [236] in 2005. The proposed strategy implies the use of energetically-activated dsDNA probes designed to target dsDNAs through dual duplex invasion, i.e., competing formation of duplexes using modified dsDNA probes to unwind and hybridize to dsDNA through Watson-Crick base paring. The strategy is considered to be attractive as it allows to target mixed-sequence dsDNAs under non-denaturating and near-physiological conditions.

**Figure 36:**
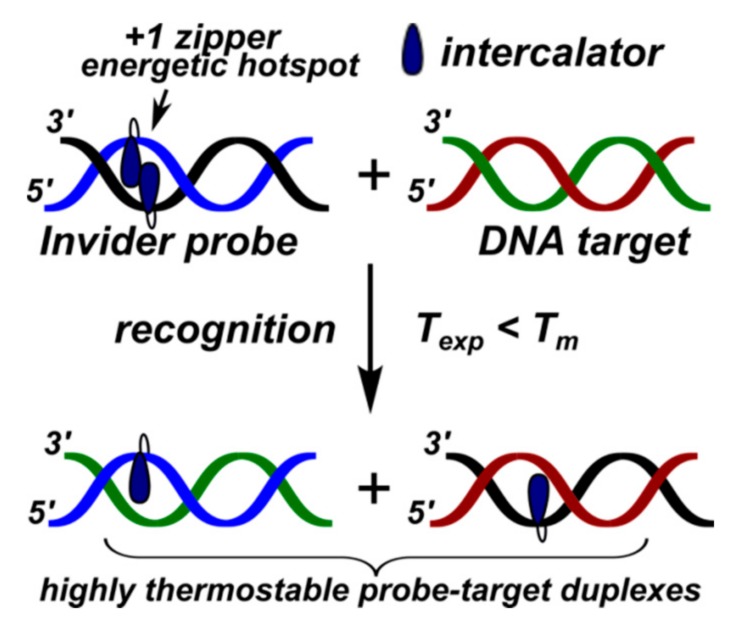
Schematic representation of the Invader probe concept for targeting of mixed-sequence dsDNAs.

Hrdlicka and coworkers have introduced so-called Invader probes, i.e., short dsDNA probes that are energetically activated through modification with one or more +1 interstrand zipper arrangements of pyrene-modified nucleotides (Figure 36) [17,56,57,58,59,60,61,62,63,237,238,239,240]. 

This +1 interstrand zipper motif forces two pyrene moieties to intercalate into the same region of the Invader probe duplex resulting in the formation of pyrene excimers and, which is more important, in unwinding and thermal destabilization of the probe (to describe the motif the authors have introduced the term “energetic hotspots”). On the other hand, both strands of the Invader probe demonstrate exceptional affinity to the target DNA due to stabilization of formed duplexes through stacking interactions of pyrene moieties and nucleobases (Δ*T*_m_/modification was up to +21 °C, varied depending on position and structure of intercalator-modified nucleotides) (Figure 36). This difference in thermal duplex stability of the double-stranded Invader probe and dsDNA target and of the resultant two probe-target duplexes, promotes the spontaneous recognition of isosequential dsDNA targets (Figure 36).

**Figure 37:**
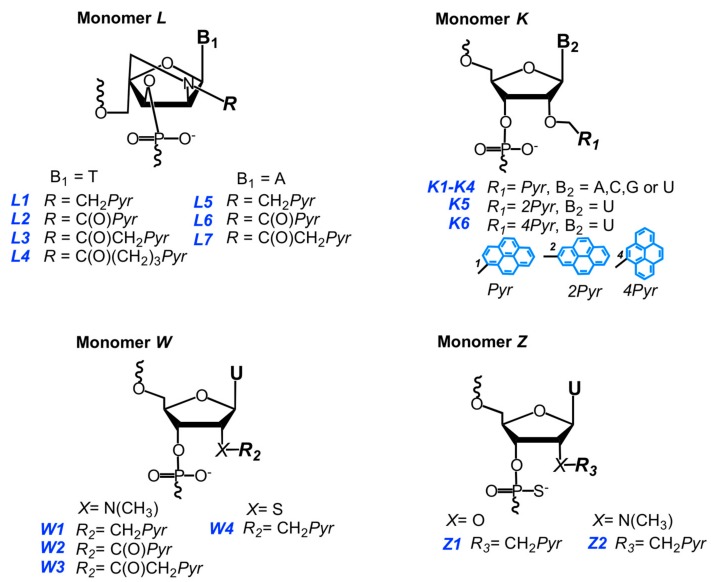
Chemical structures of pyrene-modified monomers proposed and studied as intercalator-bearing blocks of “energetic hotspots” in Invader probes. A: adenin-9-yl, U: uracil-1-yl, C: cytidin-1-yl, and G: guanin-9-yl.

Wengel, Hrdlicka, and coauthors reported *N*2′-pyrene-modified 2′-amino-α-L-LNA monomers (T [241,242] and A [243]) (monomers **L**, Figure 37). 

The first promising results have been described for Invader LNA probes, i.e., a double-stranded Invader probe modified with 2′-*N*-(pyren-1-yl)methyl-2′-amino-α-l-LNA thymine monomer (monomer **L1**, Figure 37) [17,237]. The 2′-*N*-(pyren-1-yl)methyl-2′-amino-α-l-LNA monomers, being incorporated into different positions of oligonucleotides, strongly improve the binding affinity of the probes toward DNA targets (Δ*T*_m_/modification was up to +19 °C for T [241,242] and +14 °C for A [243]). The binding of the probes to complementary targets can be monitored by the significant loss of fluorescence signal, that together with excellent thermal stabilization confirms intercalating binding mode of pyrene moiety in the DNA:DNA duplexes. The use of the constrained pyrene-modified LNA monomer provides a high degree of positional control [4], which means the pyrene moieties of these interstrand arrangements are forced to intercalate into duplex core of the probe. Invader LNA probes with one or two LNA “energetic hotspots” demonstrated fast and sequence-specific recognition of dsDNA targets in a thermal denaturation buffer (50% within ~30–50 min in medium salt buffer containing 110 mM Na^+^, pH = 7.0 at 20 °C), the recognition can be easily monitored in real time by the decrease of excimer fluorescence of the Invader LNA probe [237]. Although, due to difficult and insufficient multistep synthesis of the pyrene-modified LNA monomer **L1**, the Invader LNA probes have not been further developed. 

Later, various variants of the second generation of dsDNA Invader probes with interstrand zipper arrangements based on 2′-pyrene-modified RNA or DNA monomers were thoroughly studied. They are synthetically more readily accessible and tend to intercalate into DNA duplexes (monomers **K**, **W** and **Z**, i.e., diverse 2′-heteroatoms, linkers, affecting the orientation of intercalator or internucleotidic linkage, Figure 37) [56,57,58,59,60,61,62,63,238,239,240].

First, Hrdlicka and coworkers performed a comparative study of physico-chemical characteristics of Invader probes comprising interstrand zippers of different pyrene-modified monomers based on 2′-amino-α-l-LNA (monomers **L1**–**L7**, Figure 37), RNA (monomers **K1**, **K4**) and 2′-*N*-methyl-2′-amino-DNA (monomers **W1**–**W3**) scaffolds trying to determine structural factors responsible for successful probe activation [60]. As a result, the Invader probes with +1 interstrand zipper arrangements of monomers **K1**, **K4**, and **W1** were found to recognize dsDNA with efficiencies similar to the Invader LNA probes [60,61]. However, probes with +1 interstrand arrangements of monomer **W4** were proved to be significantly less efficient than Invader probes based on monomer **K4** or **W1** [62]. The Invader probes comprising monomers **Z1** or **Z2** with a phosphorothioate backbone are more stable towards nucleolytic degradation, although they display less efficient recognition of dsDNA targets [239].

In [57], Invader probes with three +1 interstrand zipper motifs of monomers **K1**–**K4**, have been successfully used in the 96-well plate sandwich assay as elements for two-step recognition (capture and signaling) of 28-mer dsDNA targets specific to important foodborne pathogens, namely, *Salmonella enterica*, *Campylobacter jejuni*, and *Escherichia coli*. The detection assay is characterized by high sensitivity of the specific detection of the complementary dsDNA targets at concentrations higher than ~20/30/55 pM, respectively [57]. At the same time, the possibility of the visualization of an unique region within the DYZ-1 satellite (~6 × 10^4^ repeats) on the bovine (*Bos taurus*) Y chromosome using 5′-Cy3-labeled Invader probes modified by three arrangements of monomer **K4** has been demonstrated in conditions of non-denaturating FISH experiments [56].

Then the systematic study of influence of “energetic hotspots” composition of monomers **K1**–**K4** in the probes on thermal denaturation, binding energy and recognition of dsDNA has been performed [63]. The 9-mer Invader probes comprising one hotspot of monomers **K1** and/or **K4** have shown to be the most efficient for recognition of dsDNA targets, as the resulting probe-target duplexes demonstrate the highest thermostability when modified monomers are flanked by 3′-purine nucleotides (~80% at ~100-fold excess of the probe). However, 14-mer probes with the three +1 zipper motifs demonstrate lower recognition of dsDNA (~50% at ~125-fold excess of the probe). The authors of [59] have shown that the modification of the Invader probes architecture with non-nucleosidic nonyl (C9-linker) bulge insertions leads to more affine, faster, and more persistent dsDNA recognition (relative to conventional Invader probes).

To expand further the variety of monomers that could be used as intercalating units and to study the influence of the relative orientation between the pyrene moiety and the ribose of the modified monomer, two new monomers based on 2- and 4-pyrenyl-functionalized O2′-alkylated uridine (monomers **K5** and **K6**) were obtained in [58]. Probes with “energetic hotspots” of monomer **K5** recognize the mixed-sequence dsDNA target very efficiently (slightly better than the probes with monomer **K4**). In contrast, probes based on monomer **K6** do not hybridize to the target.

More recently, the authors of [240] have suggested to merge two independent strategies for dsDNA targeting, introducing novel pseudocomplementary Invader probes, i.e., chimeras between pseudocomplementary DNA (pcDNA) and Invader probes. These chimeras are energetically activated for mixed-sequence dsDNA recognition through the introduction of pseudocomplementary base pairs (2-thiothymine and 2,6-diaminopurine) and +1 zipper motifs of monomer **W1**, respectively. The chimeras of optimal structure, where two destabilizing structural motifs were separated, demonstrated efficient recognition of mixed-sequence dsDNA targets with excellent specificity. 

Summarizing all these findings, the Invader probes are a new promising class of relatively simple probes for the targeting of unrestricted mixed-sequence dsDNAs under physiological conditions. Their potential for targeting of biologically-relevant dsDNAs in cells and mixed-sequence 28-mer DNA duplexes in sandwich assay were demonstrated. 

## 4. Supramolecular Assemblies

The ability of pyrene moieties to form stacking pairs can be a driving force for the assembly of the supramolecular oligonucleotide constructions. Indeed, the presence of pyrene fragments in the structure of an oligonucleotide provides a good precursor for nanoassemblages formed both by pyrene-pyrene stacking and complementary interactions. Häner’s group described the synthesis of oligonucleotides’ derivatives containing oligopyrene fragments either in the middle or at a terminus. The insertion was done in the process of the automated phosphoramidite oligonucleotide synthesis using pyrene phosphoramidite derivatives. 

The stacking interactions of polyaromatic fragments acts as a “molecular lock” for the stabilization of oligonucleotide complexes. Having been inserted in the middle of an oligonucleotide, they do not hinder complementary interactions; moreover, when two complementary oligonucleotides containing oligopyrene insertions located oppositely were hybridized, stacking interactions of aromatic systems significantly increased thermostability of a duplex. Strong pyrene excimer fluorescence was also observed in these duplexes [244,245]. Introduction of phenantrene moieties into the oligonucleotide structure instead of pyrene ones was demonstrated to have similar effect on the nanoconstruction formation. When pyrene and phenantrene moieties are combined in one duplex, the exciplexes formed in the stack transmit energy from one to another. Garo et al. assembled a light-harvesting antenna based on the oligonucleotide duplex, where the oligophenantrene fragment was followed by the pyrene moiety in the spacer between two complementary oligonucleotide domains. Upon the irradiation by 320 nm light (phenantrene absorbance), fluorescence energy was transmitted to the phenantrene-pyrene exciplex at the end on the stack resulting in the emission at 420 nm (emission of the exciplex) [246].

The molecular lock was also used for the stabilization of oligonucleotide tripexes. Trkulja et al. synthesized an oligonucleotide consisting of two oligopyrimidine (C, T) domains separated by the pyrene or phenantrene-containing non-nucleotide insertions and a 5′-oligopyrene-modified oligopurine (A, G) oligonucleotide forming Watson-Crick pairs with the first domain and Hoogsteen pairs with the second domain. When hybridized, ologonucleotides form a triplex structure; meanwhile, the terminal pyrene moiety is stacking with the oligopyrene or oligophenantrene spacer. This results in the large increase of the melting temperature of the triplex in comparison with that of control complexes without polyaromatic modifications, with the pyrene-phenantrene pair stabilizing the structure stronger than pyrene-pyrene pair. The emission of the pyrene-phenantrene exciplex in the triplex structure was stronger and red-shifted in comparison with the emission of the pyrene excimer [247]. Later, the pyrene-phenantrene and pyrene-pyrene stacking was used to assemble an oligonucleotide construction containing two triplexes. Two oligonucleotides were designed to have an oligopyrimidine and an oligopurine complementary domains separated by the T4 spacer, as well as a triplex-forming oligopyrimidine domain introduced via the spacer containing polyaromatic insertion. When the hybridization occurs, complementary domains of the first oligonucleotide form a duplex, whereas the triplex-forming domain remains unpaired. The interaction with the second oligonucleotide provokes the formation of two triplexes, the whole construction is stabilized by the stacking of polyaromatic moieties. There was just a slight difference between melting temperatures of the complexes stabilized by pyrene-phenantrene and pyrene-pyrene pairs [248].

**Figure 38:**
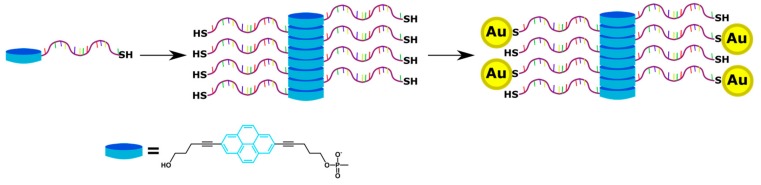
Pyrene-driven assembly of oligonucleotides’ conjugates into nanorods and the subsequent grafting of gold nanoparticles [253].

It should be noted that the stacking interactions of oligopyrene fragments can define the formation of oligonucleotide nanoconstructions entirely. When introduced at the oligonucleotide termini, oligopyrene fragments of different molecules interact in solution to form polypyrene [249,250] or mixed polypyrene-perylenediimide [251,252] stacks retaining oligonucleotide parts anchored. These rod-shaped nanoassemblies are quite stable and are decomposed to the monomers only after heating. The interaction of such nanoassemblies containing complementary oligonucleotides leads to the pairing-driven formation of nanorod networks (Figure 38), as confirmed by AFM [249,250]. The oligonucleotide nanorods were also decorated with gold nanoparticles. To do this, oligonucleotides bearing oligopyrene fragments and thiol groups at the opposite termini were used as precursors for nanoassemblies. When nanorods have been formed, gold nanoparticles were deposited on them through the interactions with thiols [253]. Thus, the nanoassemblies can serve as a platform for the functional nanoconstructions.

The data presented herein suggest that the pyrene-modified oligonucleotides are prospective building blocks for nanoarchitecture. The combination of two functional fragments provoking the self-assembly can lead to the nanomaterials with a precisely controllable structure. Pyrene-modified oligonucleotides can be a component of complex hybrid nanoconstructions. In particular, pyrene can be is used for the immobilization of oligonucleotides on the surface of sp^2^-carbon nanostructures: carbon nanotubes and graphene. When conjugated with a cargo, the pyrene moiety provides its anchoring on a carbon surface. The π-π stacking interactions that drive the anchoring are irreversible at standard and physiological conditions and stable towards the displacement with surfactants, biomolecules, and most part of polyaromatic compounds [20,21]. These properties make carbon nanoparticles bearing anchored oligonucleotides prospective tools for molecular diagnostics and therapeutics. The first example of the non-covalent hybrids of oligonucleotides with carbon nanotubes (CNTs) obtained through pyrene anchoring was reported by Taft et al. [254]. Conjugates of 5′-amino-terminated DNA oligonucleotides with 1-pyrenebutanoic acid were adsorbed on the surface of arrayed multi-walled carbon nanotubes (MWCNTs). The immobilization was not observed to affect significantly the hybridization ability of oligonucleotides: after the anchoring, the oligonucleotides were hybridized with complementary oligonucleotides appended to gold nanoparticles to form the three-layer hybrid material.

**Figure 39:**
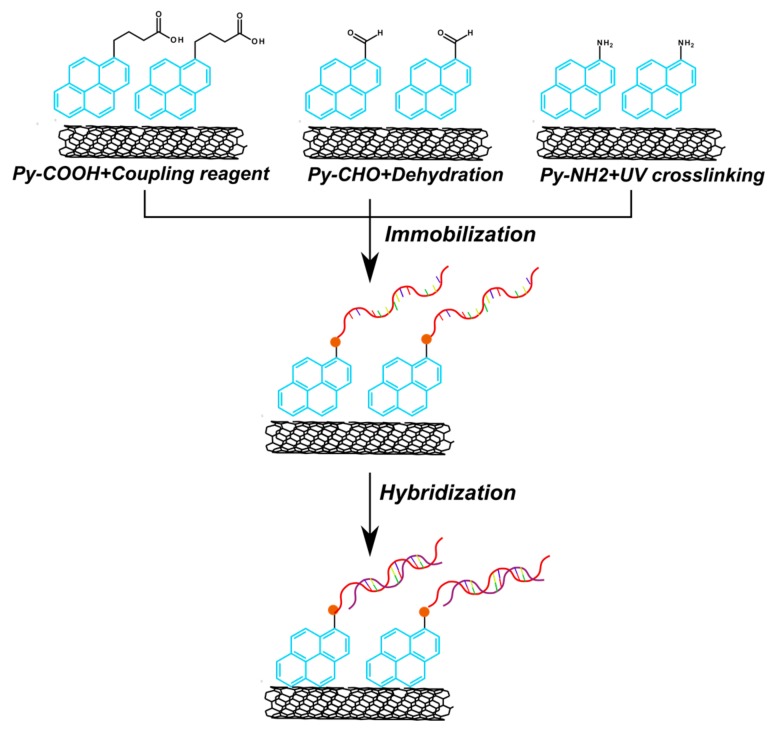
The procedure for the immobilization of a DNA probe on the pyrene-functionalized SWCNTs and the subsequent process for the hybridization of target DNAs.

The leading role in the anchoring of oligonucleotides on the CNT surface belongs to pyrene [255]. An oligonucleotide immobilized is able to interact with a complementary oligonucleotide [256]. This property was used for the design of CNT-based devices for biosensing. Joiner et al. [257] designed a field-effect transistor device where a film of individual single-walled carbon nanotubes (SWCNTs) was deposited onto the electrical circuit and acted as a transducer for the sensor. The surface of SWCNTs was modified with 1-pyrenebutanoic acid *N*-oxysuccinimide ester, and then 5′-amino-modified oligonucleotide was grafted to the construction. The detection of a complementary target was done by conductometry; the electroactive intercalators were used as signal amplifiers. The biosensor described efficiently discriminated a complementary and a mismatch-containing targets. Baek et al. studied the effect of the structure of the pyrene anchor on the biosensor performance [258]. Three types of pyrene derivatives (1-pyrenebutanoic acid, 1-pyrene carboxaldehyde, and 1-pyrenyl methylamine) were adsorbed on SWCNT films and reacted with oligonucleotides in specific conditions (carboxylic group activation, Schiff’s base reaction, followed by the reduction of amide, and UV-crosslinking, respectively) (Figure 39). The performance of the label-free discrimination between complementary and non-complementary oligonucleotide targets was assessed by means of fluorometry and voltammetry. The use of 1-pyrenebutanoic acid as an anchor group was shown to provide the best selectivity and sensitivity of the biosensor. However, Fedorovskaya et al. have assembled electrodes based on vertically-aligned MWCNT arrays functionalized with 5′-1-pyrenylmethylaminophosphate-modified RNA oligonucleotides and demonstrated significant differences between cyclic voltammetry profiles of the electrodes in the presence of complementary and non-complementary target oligonucleotides [259]. It should be noted that graphene is also an appropriate support for the immobilization of pyrene-modified oligonucleotide probes. Zhang et al. designed graphene-coated gold electrode and functionalized it with 1-pyrenebutanoic acid *N*-oxysuccinimide ester, followed by the grafting of the 5′-amino-modified oligonucleotide probe [260]. Cyclic voltammetry and electron impedance spectroscopy demonstrated high sensitivity of the biosensor towards complementary target in the presence of the electroactive indicators, methylene blue, or [Fe(CN)_6_]^3−/4−^ (the limit of detection was 380 aM).

Protein targets can also be bound by DNA and RNA aptamers anchored on CNTs by means of pyrene moieties [256]. Maehashi et al. used the abovementioned methodology to design an IgE aptasensor [261,262]. The recognition of a protein target was done by the 5′-pyrene-modified 45-nucleotide DNA aptamer that was grafted to the SWCNTs. The detection limit of the aptasensor was 250 pM. In order to detect the IgE, two kinds of receptor (monoclonal IgE antibody and anti-IgE aptamer)-modified CNT-FET devices were fabricated by Kim et al. [263]. The results show that CNT-FET biosensors using monoclonal IgE antibody had very low sensitivity (minimum detectable level ~1000 ng/mL), while those based on the anti-IgE aptamer could detect 50 ng/mL. Khosravi et al. reported a similar conductometric aptasensor of interleukin-6 having a sensitivity of 1 pg/mL [264]. A 32-nucleotide RNA aptamer was grafted to the SWCNT microarray modified with 1-pyrenebutanoic acid *N*-oxysuccinimide ester and used as a recognition element. 

**Figure 40:**
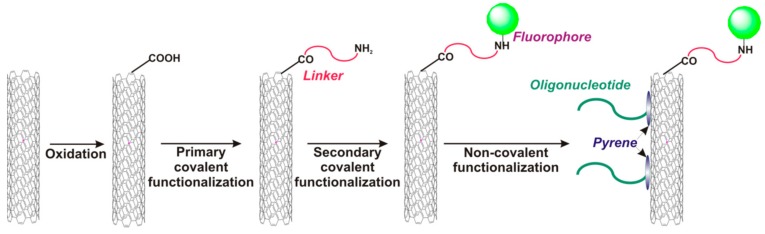
Strategy of the synthesis of multifunctional hybrids of carbon nanotubes (CNTs) with oligonucleotides.

Along with the use of pyrene-modified oligonucleotides in hybrids with carbon nanotubes as recogntition elements, their role in the design of nanocarbon-based transfection agents should also be mentioned. Indeed, since pyrene is a convenient moiety to immobilize nucleic acid fragments on the surface of sp^2^-hybrid carbon nanoparticles, it can be used for the grafting of therapeutically-relevant oligonucleotides to the carrier constructions. In particular, carbon nanotubes, both SWCNTs and MWCNTs, are efficient carriers for different types of nucleic acid therapeutics [265]. Apartsin et al. proposed the use of pyrene-modified oligonucleotides to assemble multifunctional hybrids of nucleic acids with carbon nanotubes through two orthogonal functionalization stages. Highly-dispersed SWCNTs were covalently functionalized with biomimetic moieties on the tips and defect sites of the surface, then the defectless surface was non-covalently functionalized with 5′-pyrene conjugates of DNA, RNA, or 2′-*O*-methyl RNA oligonucleotides (Figure 40). The efficiency of the oligonucleotides’ adsorption of the CNT surface did not depend on the nature of oligonucleotides [266]; however, it could be increased by the introduction of a long flexible linker (oligoethylene glycol) between pyrene and oligonucleotide [267]. Anchored oligonucleotides remain available for the interaction with cellular metabolites and can be released from the hybrid, if a biodegradable bond is introduced in the linker [268]. Multifunctional hybrids were obtained based on the SWCNTs functionalized with aliphatic amines [269,270], diamino-polyethylene glycol [268], and cationic PAMAM dendrimer [269]. The hybrids efficiently penetrated into a cell by means of endocytosis; however, no cytotoxic and genotoxic effects of hybrids were shown in therapeutically relevant concentrations [268].

## 5. Miscellaneous Applications

### 5.1. Pyrene-Mediated Photolysis of Disulfide Bonds in Oligonucleotides 

**Figure 41:**
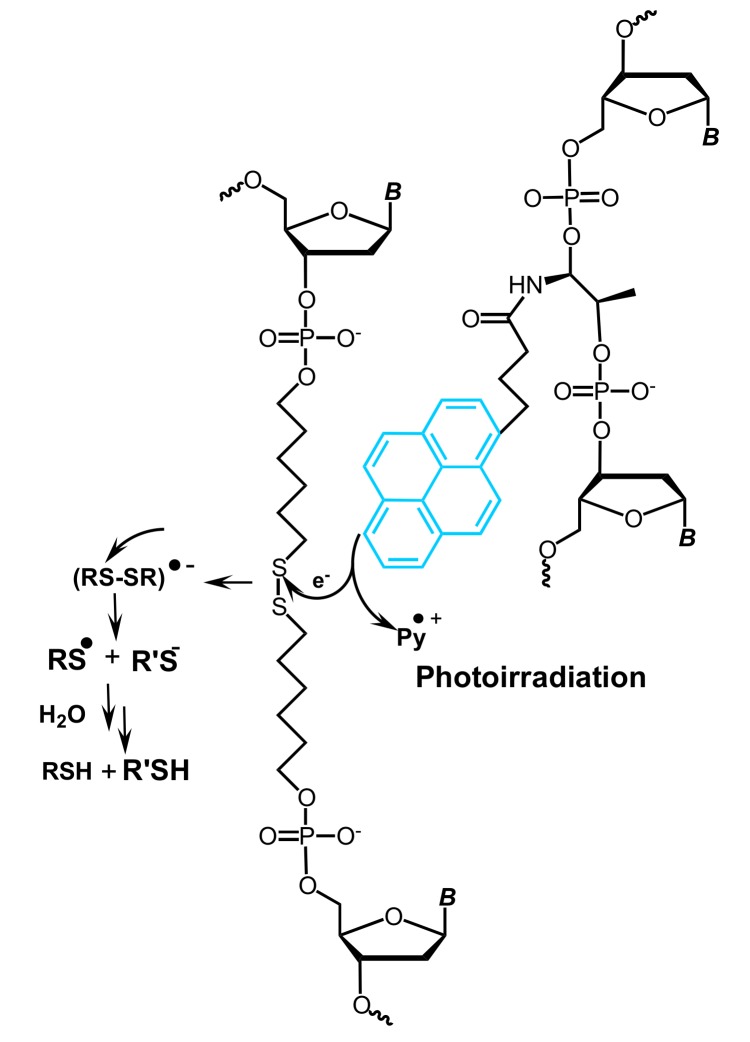
Pyrene-assisted photolysis of disulfide within DNA structure.

In 2010, Tan et al. discovered that the pyrene moiety, introduced into the oligonucleotide backbone as a non-nucleoside insertion, enhances the photolysis of disulfide bonds in its vicinity when irradiated by UV light at the pyrene-specific wavelength [271] (Figure 41).

The provisional mechanism of the photolysis presumes the formation of pyrene cation-radical that facilitates the formation of the disulfide anion-radical which further decomposes to form two thiol species, a radical and an anion. The phenomenon observed was used for the design of photolabile supramolecular assemblies. Oligonucleotides containing pyrene in a non-nucleotide insertion at the 5′-termini were conjugated with lipophilic moieties through a disulfide bond. Such conjugates efficiently formed micelles in water. The irradiation of these micelles by 350 nm light caused the photolysis of disulfide bonds, resulting in the decomposition of nanoconstructions.

When introduced in the middle of a DNAzyme, the pyrene moiety irradiated by the UV light was demonstrated to cause the cleavage of a DNA substrate consisting of two oligonucleotides linked through a disulfide bond. Such a DNAzyme cleaves the substrate in the catalytic regime; its properties (K_M_ and k_cat_) are comparable to those of classic ribozymes [271]. These findings were used to develop a fluorescent biosensor system for the detection of Hg^2+^ ions [272]. Two T-rich oligonucleotides were synthesized: the first one contained a pyrene moiety in the middle, and the second one consisted of two domains linked by the FAM-disulfide-Dabcyl fragment. Two oligonucleotides were assembled in the complex in the presence of Hg^2+^ ions. In the complex, pyrene caused the cleavage of the disulfide upon irradiation followed by the disruption of the complex and release of the FAM- and Dabcyl-containing domains resulting in the increase of the FAM fluorescence. The sensing system had high selectivity of the Hg^2+^ recognition over the competing ions of other metals. 

Pyrene-mediated photolysis of disulfide bonds can be a driving force for the functioning of molecular machines. Tan’s group constructed a DNA track, a single-stranded DNA oligonucleotide, complexed with four anchoring sequences—short oligonucleotides partially hybridized with the track and containing a disulfide linkage in the middle of the non-hybridized part. The moving part, a DNA walker, is an oligonucleotide bearing a pyrene moiety between two fragments (motion legs). The walker oligonucleotide is complexed with the non-hybridized fragment of the anchoring oligonucleotide, with the pyrene being positioned facing the disulfide bond. Upon UV irradiation, this bond is cleaved. The affinity of the walker greatly decreases, and it is hybridized with the next anchoring oligonucleotide, part by part. By repeating these stages, the light-directed molecular motion is achieved [273]. In the later work, similar molecular machine was assembled on a 2D DNA tile produced by means of a DNA origami technique. The organization of the machine on the surface permitted performing the direct visualization of the walking using high-speed atomic force microscopy [274].

### 5.2. Pyrene as a Structure Element of RNA Cleaving and Abasic Site Recognizing NA Constructions 

RNA-cleaving nucleic acid constructions are valuable tools to inhibit the expression of a target gene in a sequence-specific manner, and may be used for functional genomics, target validation, and therapeutic purposes. Catalytic nucleic acids (CNAs) have provided scientists with valuable tools for new therapies through downregulation or modulation of gene expression [275,276,277]. Ribozymes, a class of CNAs, can be mostly used to down-regulate (by RNA cleavage) unwanted gene expression involved in disease. Deoxyribozymes or DNAzymes are single-stranded catalytic DNA molecules that are obtained by combinatorial in vitro selection methods. DNAzymes are also able to bind and to cleave RNA targets and, therefore, down-regulate gene expression [276,277,278].

**Figure 42:**
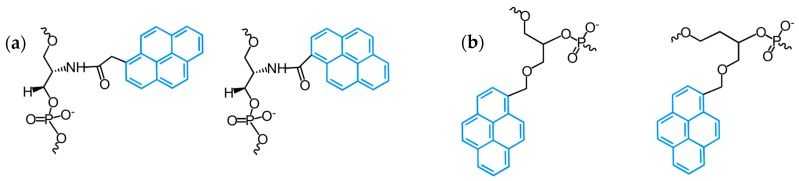
Structure of pyrene monomers used as structure elements of different nucleic acid (NA)-constructions. (**a**) Pyrene monomers for DNAzyme catalytic core modification [279,280]; and (**b**) pyrene linkers for bulge loop stabilization [282].

The activity of RNA-cleaving 10–23 DNAzyme was improved by covalently introducing pyrene into the catalytic core [279,280]. Pyrene was attached to D-threoninol via amide bond, inserted into the DNA backbone through routine phosphoramidite chemistry (Figure 42a). Highly active variants of minimal hammerhead ribozymes were generated by the replacement of substantial parts of stem-loop structures with pyrene building blocks [281] (**Pyrene-linker**, Figure 31).

A pyrene-modified linker was incorporated into the DNA strand to facilitate the formation of a RNA bulge loop in the RNA/DNA duplex [282] (Figure 42b). The pyrene-stabilized bulge loop in HIV-1 *TAR* RNA was efficiently and site-specifically cleaved by trans-(±)-cyclohexane-1,2-diamine. 

Pyrene monomer **Y** as a selective element for abasic site recognition was used in [283] (Figure 29). An RNA abasic site was generated by treatment of an RNA hairpin structure by Ricin Toxin A-Chain (RTA) and then recognized by pyrene-modified oligonucleotide.

The data presented in these parts of the review (Section 4 and Section 5) suggest, once more, the many-sided possibilities of applications of pyrene-modified oligonucleotides.

## 6. Conclusions

The wide spectra of publications devoted to recent advances in nucleic acid targeting and supramolecular assembly using pyrene-modified oligonucleotides discussed herein and in other related reviews [4,7,9,10,33,34,35,64] confirm their increasing all-round potential for applications in molecular biology studies, diagnostics, and nanotechnology. Although significant progress has been made in the approaches utilizing pyrene-modified oligonucleotides, there are several remaining challenges that should be addressed in the future. For instance, the development of ultrasensitive fluorescent systems, suitable for direct detection of NA targets in vitro and in vivo with low detection limits and sensitivity to SNPs, is still of great importance. In view of the progress made and the latest advances in various signal amplification methods (see, for instance, recent reviews [284,285]), we believe, this task can be solved. Further development of the sequence-specific dsDNA targeting agents possessing increased selectivity and affinity to the target under near physiological conditions, and an enhanced ability to pass through the cell membrane is also required. These agents would become valuable tools for a range of in vivo applications, such as regulation of the gene expression via inhibition of transcription, correction of mutations in DNA, and visualization of chromosomal DNA in living cells. Moreover, it is very likely that the future of the pyrene-modified oligonucleotides will be related to better understanding and following modulation of their properties as key components of more sophisticated systems, e.g., sensing devices based on DNA multichromophoric systems, DNA-based light-harvesting antennas, NA-based molecular machines, and self-assembled functional nanomaterials.
